# Roles and Mechanisms of Gut Microbiota in Patients With Alzheimer’s Disease

**DOI:** 10.3389/fnagi.2021.650047

**Published:** 2021-05-28

**Authors:** Shaochang Wu, Xia Liu, Ruilai Jiang, Xiumei Yan, Zongxin Ling

**Affiliations:** ^1^Department of Geriatrics, Lishui Second People’s Hospital, Lishui, China; ^2^Department of Intensive Care Unit, The First Affiliated Hospital, School of Medicine, Zhejiang University, Hangzhou, China; ^3^Collaborative Innovation Center for Diagnosis and Treatment of Infectious Diseases, State Key Laboratory for Diagnosis and Treatment of Infectious Diseases, National Clinical Research Center for Infectious Diseases, The First Affiliated Hospital, School of Medicine, Zhejiang University, Hangzhou, China; ^4^Institute of Microbe & Host Health, Linyi University, Linyi, China

**Keywords:** Alzheimer’s disease, gut-brain axis, microbiota, probiotics, short chain fatty acids

## Abstract

Alzheimer’s disease (AD) is the most common age-related progressive neurodegenerative disease, characterized by a decline in cognitive function and neuronal loss, and is caused by several factors. Numerous clinical and experimental studies have suggested the involvement of gut microbiota dysbiosis in patients with AD. The altered gut microbiota can influence brain function and behavior through the microbiota–gut–brain axis via various pathways such as increased amyloid-β deposits and tau phosphorylation, neuroinflammation, metabolic dysfunctions, and chronic oxidative stress. With no current effective therapy to cure AD, gut microbiota modulation may be a promising therapeutic option to prevent or delay the onset of AD or counteract its progression. Our present review summarizes the alterations in the gut microbiota in patients with AD, the pathogenetic roles and mechanisms of gut microbiota in AD, and gut microbiota–targeted therapies for AD. Understanding the roles and mechanisms between gut microbiota and AD will help decipher the pathogenesis of AD from novel perspectives and shed light on novel therapeutic strategies for AD.

## Introduction

Alzheimer’s disease (AD), the most common form of age-related dementia, is characterized clinically by insidious onset of memory and cognitive impairment, emergence of psychiatric symptoms and behavioral disorders, and impairment of activities of daily living (Scheltens et al., 2021). The early clinical symptoms of AD are nonspecific including changes in thinking or unconscious behavior, memory impairment with respect to new information, and dysfunctional changes in language and speech, while its consequences are fatal and irreversible at the time of diagnosis. Current therapies for AD are only available for symptomatic relief, but thus far, no treatment is available to delay or reverse disease progression. AD is recognized as a common cause of an estimated 60–80% of cases of dementia, ranking as the sixth leading cause of death in the United States (Alzheimer’s Association, 2018). As an age-related progressive neurodegenerative disease, the prevalence of AD is rapidly increasing worldwide especially among the elderly (age > 60 years). According to current statistics, nearly 50 million people worldwide suffer from AD or AD-related dementia (Alzheimer’s Association, 2018). Based on a national survey conducted by the National Bureau of Statistics, China’s elderly population reached 254 million at the end of 2019, making up about 18.1% of the total population, and by 2050, nearly 35% will be aged 60 years or older. With the rapid increase in the aged population, 10 million Chinese people have lived with AD, and the number will exceed to 30 million by 2050, ranking first in AD cases in the world. The annual socioeconomic cost per AD patient was United States $19,144.36, and the total cost was United States $167.74 billion in 2015. The annual total costs are predicted to reach United States $507.49 billion in 2030 and United States $1.89 trillion in 2050 (Jia et al., 2018). Taken together, the above statistics indicate that AD not only affects morbidity or mortality but also affects the socioeconomic and health care burden in China (Jia et al., 2018; Cui et al., 2020). Although tremendous efforts have been made to treat AD, no efficient disease-modifying therapeutics are available, partially because of the limited understanding of the disease pathogenesis.

Numerous studies in recent decades have focused on elucidating the etiopathology of AD, but its pathogenesis remains unclear, and no therapeutic strategy is available to cure this disease. Various molecular, biochemical, and cellular abnormalities such as cell loss, impaired energy metabolism, increased activation of signaling pathways, amyloid-β (Aβ) deposits, mitochondrial dysfunction, chronic oxidative stress, impaired energy metabolism, and DNA damage are involved in the pathogenesis of AD (Guo et al., 2020; Zhang Y. X. et al., 2020; Zolochevska and Taglialatela, 2020; Ashford et al., 2021). The neuropathological hallmarks of AD include the formation of senile plaques and neurofibrillary tangles in specific brain regions that lead to synaptic loss and neuronal death (Khan and Hegde, 2020; Mattsson-Carlgren et al., 2020; Konijnenberg et al., 2021). Since 1984, the “amyloid hypothesis” of AD has continued to gain support, particularly from genetic studies (Glenner and Wong, 1984). Extracellular Aβ deposition (including Aβ40 and Aβ42), produced by protease cleavage of the type I transmembrane amyloid precursor protein (APP), can cause secondary pathological changes such as hyperphosphorylation of tau (p-tau), neuroinflammation, oxidative stress, and neurite degeneration, eventually leading to AD (Hardy and Selkoe, 2002). Compelling evidence suggests that Aβ deposition can activate microglia and recruit astrocytes, leading to a local inflammatory response, which may contribute to neuronal degeneration and cell death. In the last few decades, studies have provided growing evidence for a central role of Aβ and tau as well as glial contributions to various molecular and cellular pathways in AD pathogenesis (Guo et al., 2020). Accumulation of Aβ fibrils is thought to be an initiating factor in AD and necessary for the formation of tau aggregates (Goedert, 2018). Normal tau is essential for neuron extension configuration, cell polarization, and axonal transport, while hyperphosphorylation of tau leads to instability of the cytoskeleton, collapse of microtubules, synaptic failure, impairment of communication, and neuronal death (Zhang M. et al., 2020). Recently, the link between Aβ and tau in AD has been clarified. Mattsson-Carlgren et al. (2020) found that Aβ deposition is associated with increases in the levels of soluble and phosphorylated tau that precede positive tau positron emission tomography (PET) in AD. Johnson et al. (2016) demonstrated that quantitative tau by PET has been shown to correlate with cognitive performance in AD even more robustly than Aβ. Cerebrospinal fluid (CSF) Aβ and tau can target and disrupt synapses thus driving cognitive decay, which can be used as biomarkers for AD diagnosis (Holtzman et al., 2011). These CSF and imaging biomarkers combined with several relatively new clinical criteria can aid diagnosis in living patients (Ritchie et al., 2017; Weller and Budson, 2018; Leuzy et al., 2021); however, the definitive diagnosis of AD still requires postmortem evaluation of brain tissue.

Alzheimer’s disease is a multifactorial neurodegenerative disease caused by several critical factors including genetic and non-inheritable components such as aging, variables related to the environment, lifestyle habits, and describe the potential role of apolipoprotein E, viral and bacterial infection, insomnia, and gut microbiota (Guo et al., 2020). Although genetics has been described to play the most important role in the etiology of AD, environmental factors such as gut microbiota are also strongly believed to contribute to their development and/or manifestation (Cenit et al., 2017). Gut microbiota composition has been shown to contribute to the regulation of social behavior, stress resistance, and cognitive functions (Foster and McVey Neufeld, 2013). Heijtza et al. (2011) reported that germ-free mice display reduced anxiety-like behavior in tests such as the light/dark test. Previous clinical studies have suggested that individuals with microbial dysbiosis caused by intestinal diseases are at high risk to develop AD (Caini et al., 2016; Chen et al., 2016). Recent studies have focused on gut microbiota alterations and their possible impact on brain function and neurodegenerative diseases such as AD (Cattaneo et al., 2017; Vogt et al., 2017; Zhuang et al., 2018; Li B. et al., 2019; Liu P. et al., 2019; Ling et al., 2021a,b). Mounting evidence suggests that gut microbiota influences not only gastrointestinal physiology but also brain function and behavior (Diaz Heijtz et al., 2011; Desbonnet et al., 2015). There is a bidirectional relationship between the brain, gut, and gut microbiota, which is referred to as the microbiota–gut–brain axis (Cryan and Dinan, 2012; Burokas et al., 2015). The widely accepted concept of “microbiota–gut–brain axis” has been established for nearly 15 years, which can help decipher the neurobiological mechanisms underpinning the influence exerted by gut microbiota on brain function and behavior. Currently, a body of preclinical and to a lesser extent epidemiological evidence supports the notion that host–microbiota interactions play a vital role in brain development and function and in the etiology of neurodevelopmental disorders. The human gut microbiota comprise an enormous number of microorganisms, nearly 100 times larger than our own cells, and play crucial roles in human development, physiology, immunity, and nutrition, sometimes called the “second brain” (Dinan and Cryan, 2017; Sochocka et al., 2019). Continuous dynamic cross-talk between the gut and the brain is facilitated by neuronal, endocrine, metabolic, and immune pathways (Morais et al., 2021; Wilmes et al., 2021), which have been considered as the biological and physiological basis of psychiatric, neurodevelopmental, age-related, and neurodegenerative disorders such as AD. Gut microbiota and the brain communicate with each other via various routes which include: (i) regulating immune activity and the production of proinflammatory cytokines (e.g., IL-1β, IL-6, and IL-17A) or anti-inflammatory cytokines (e.g., IL-4 and IL-10) that can either stimulate the hypothalamic-pituitary-adrenal axis to produce corticotropin-releasing hormone, adrenocorticotropin hormone and cortisol, or directly impact on central nervous system (CNS) immune activity; (ii) production of short-chain fatty acids (SCFAs), branched chain amino acids, and peptidoglycans; (iii) the production of neurotransmitters (e.g., acetylcholine, GABA, dopamine, and serotonin) that may enter circulation and cross the blood-brain barrier; (iv) modulating tryptophan metabolism and downstream metabolites, serotonin, kynurenic acid and quinolinic acid; (v) affecting vagus nerve and the enteric nervous system; (vi) impacting enterochromaffin cells by SCFAs and indole (Kennedy et al., 2017; Cryan et al., 2019; Morais et al., 2021). These mechanisms can mediate gut microbiota by actively participating in the development of various brain disorders, including AD. Studies in germ-free animals and animals exposed to pathogenic microbial infections, antibiotics, probiotics, or fecal microbiota transplantation (FMT) have shown the role of gut microbiota in host cognition or pathogenesis related to AD (Jiang et al., 2017). Microbiota-gut-brain axis signaling has uncovered a new era in psychiatry that is expected to provide novel targets for the diagnosis and treatment of psychiatric disorders and decipher their pathogeneses. A current novel tenet supports the idea that AD pathogenesis is not only closely related to gut microbiome imbalance but may also originate in the gut. In the present review, we will discuss the alterations in the gut microbiota in patients with AD, the pathogenetic roles and mechanisms of gut microbiota in AD, and gut microbiota-targeted therapy for AD. Understanding the roles and mechanisms between gut microbiota and AD will help decipher the pathogenesis of AD from novel perspectives and shed light on novel therapeutic strategies for AD in the future.

## Alterations in Gut Microbiota in Patients With AD

The human body, consisting of 10^13^ human cells and 10^14^ commensal microbiota (the revised estimates placing the ratio of human to bacteria cells to an approximate 1:1 ratio), is considered as a human superorganism whose integrity is pivotal for health (Sender et al., 2016). Approximately 95% of commensal microorganisms in the human body are located in the gut. The human gut microbiota is influenced by multiple and diverse factors, including physiological factors (age, ethnicity, origin, and environment) and others linked to external factors, such as hygiene, dietary habits, antibiotics, and probiotics (Guarner, 2007; Ziegler-Graham et al., 2008). Gut microbiota has been identified and proposed to be a key modulator of human health, to the extent that it has been proposed to be an “essential organ” of the human body. Gut microbiota is involved in important homeostatic processes, not only related to gastrointestinal function but also to several complex modulatory processes, such as glucose and bone metabolism, inflammation and immune response, and peripheral (enteric), and central neurotransmission (De-Paula et al., 2018). Growing evidence has revealed that gut microbiota participates actively in the development of obesity, diabetes, cancers, aging, autoimmune diseases and even neuropsychiatric disorders such as depression and AD (Jiang et al., 2015; Zhang et al., 2015; Cattaneo et al., 2017; Zhao et al., 2018; Li B. et al., 2019; Ling et al., 2019, 2021a,b; Liu X. et al., 2019; Liu X. et al., 2020; Cheng et al., 2020; Sun et al., 2021). As mentioned by Cani, gut microbiota may be “at the intersection of everything,” being implicated in virtually all physiological or pathological situations (Cani, 2017). Technological improvements, especially multi-omics techniques, have facilitated the growth of microbiota research and the rapid expansion of knowledge over the past few years has helped to reinforce our understanding of gut microbiota and AD (Gilbert et al., 2018). Currently, researchers have paid more attention to gut microbiota research on neurochemistry, neurophysiology and neuropsychiatry. More recently, several clinical studies have proposed that dysbiosis of gut microbiota is a key factor that influences brain function and behavior through the microbiota–gut–brain axis, leading to the development of AD (Cattaneo et al., 2017; Vogt et al., 2017; Zhuang et al., 2018; Haran et al., 2019; Li B. et al., 2019; Liu P. et al., 2019; Guo et al., 2021; Ling et al., 2021a,b). Morris et al. (2017) reported that 85% of patients with dementia have different gut microbiota compositions from the healthy population. Although there are significant interpersonal variations, gut dysbiosis, especially several specific bacterial taxa, has been found to be involved in the pathogenesis of AD. Several observational studies mentioned above, most case-control designs, investigated the relationship between gut microbiota and AD in different countries and ethnicities (Table 1). Although geography exerts a strong effect on human gut microbiota variations (He et al., 2018), compelling evidence indicates that pathological changes in gut microbiota play vital roles in modulating the microbiota–gut–brain axis and actively participate in the etiopathogenesis of AD. Vogt et al. (2017) first performed a comprehensive survey with bacterial 16S rRNA gene sequencing to characterize the bacterial taxonomic composition of fecal samples from United States participants with and without a diagnosis of dementia because of AD. They discovered that the microbiome of patients with AD has decreased microbial richness and diversity and a distinct composition compared to asymptomatic age- and sex-matched control participants. The phylum abundance of Firmicutes and Actinobacteria decreased significantly, whereas Bacteroidetes were enriched in patients with AD. At the genus level, *Bifidobacterium*, SMB53 (family Clostridiaceae), *Dialister*, *Clostridium*, *Turicibacter*, and cc115 (family Erysipelotrichaceae) were all less abundant, whereas *Blautia*, *Phascolarctobacterium*, *Gemella, Bacteroides, Alistipes* and *Bilophila* were all more abundant in participants with ADs. Interestingly, the levels of these differential key functional bacteria correlated with CSF biomarkers of AD pathology such as Aβ_42_/Aβ_40_, p-tau and p-tau/Aβ_42_, which suggested that the altered AD microbiome can link the neuropathological changes in AD. Another Italian study conducted by Cattaneo et al. (2017) enrolled 10 cognitively healthy amyloid-negative controls, 40 cognitively impaired amyloid-positive patients, and 33 cognitively impaired amyloid-negative patients. They measured the abundance of selected bacterial taxa using stool samples in gut microbiota, including *Escherichia/Shigella*, *Pseudomonas aeruginosa*, *Eubacterium rectale*, *E. hallii*, *Faecalibacterium prausnitzii*, and *Bacteroides fragilis* with quantitative PCR. A lower abundance of *E. rectale* and a higher abundance of *Escherichia/Shigella* were found in amyloid-positive patients compared to both healthy controls and amyloid-negative patients. A positive correlation was observed between pro-inflammatory cytokines IL-1β, NLRP3, and CXCL2 with abundance of the inflammatory bacteria taxon *Escherichia/Shigella* and a negative correlation with the anti-inflammatory *E. rectale*. This study provided direct evidence that gut microbiota composition may drive peripheral inflammation, contributing to brain amyloidosis and, possibly, neurodegeneration and cognitive symptoms in AD. Another United States clinical study enrolled 108 nursing home elders (51 no dementia, 24 AD elders and 33 other dementia types) and were followed up for up to 5 months; longitudinal stool samples were collected for metagenomic analysis and *in vitro* T84 intestinal epithelial cell functional assays for P-glycoprotein expression, a critical mediator of intestinal homeostasis (Haran et al., 2019). They identified numerous microbial taxa (increased abundance of: *Bacteroides* spp., *Alistipes* spp., *Odoribacter* spp. and *Barnesiella* spp.; decreased: *Lachnoclostridium* spp.) and functional genes that act as predictors of AD dementia in comparison to older adults without dementia or with other dementia types, which could induce lower P-glycoprotein expression levels *in vitro*. The AD microbiome is characterized by a lower proportion and prevalence of bacteria with the potential to synthesize butyrate such as members of the *Butyrivibrio* (*B. hungatei* and *B. proteoclasticus*) and *Eubacterium* (*E. eligens*, *E. hallii*, and *E. rectale*), *Clostridium* sp. strain SY8519, *Roseburia hominis*, and *F. prausnitzii*, as well as higher abundance of taxa that are known to cause proinflammatory states including *Odoribacter splanchnicus* and *Bacteroides vulgatus*. Machine learning approaches that combine both metagenomic and clinical data, they have demonstrated that the AD microbiome can affect intestinal health via dysregulation of the P-glycoprotein pathway. This work provides an important advance to bridge the gap in how the AD microbiome can potentially adversely and affect intestinal epithelial homeostasis via dysregulation of the P-glycoprotein pathway.

**TABLE 1:** Clinical microbiome studies in Alzheimer’s disease.

**Authors/location**	**Year**	**Sample size**	**Sequencing platform**	**Result**
Vogt et al. United States	2017	Primary compositional analysis: 25 AD patients and 25 controls; Secondary CSF correlational analysis: 9 AD patients, and 31 non-demented ones.	Miseq/16S rRNA V4 regions for fecal samples	1 ACE and Chao1 ↓; Shannon and Faith’s Phylogenetic Diversity↓; Compositional difference in PCoA. 2 Phylum level: Firmicutes and Actinobacteria ↓, Bacteroidetes ↑. 3 Genera level: *Bifidobacterium*, SMB53, Dialister, Clostridium, Turicibacter, Adlercreutzia, cc115 ↓; Blautia, Bacteroides, Alistipes, Phascolarctobacterium, Bilophila, Gemella ↑. 4 PiCRUSt: Metabolism and biosynthesis ↑, signal transduction and cell motility ↓. 5 Correlation analysis: Differentially abundant genera and CSF biomarkers (Aβ42/Aβ40, p-tau, p-tau/Aβ42) of AD.
Cattaneo et al. Italy	2017	40 amyloid-positive, 33 amyloid-negative, 10 healthy controls	Quantitative PCR	1 *Lower Eubacterium rectale and higher Escherichia/Shigella in* amyloid-positive patients *compared to both healthy controls and* amyloid-negative patients. 2 *A positive correlation between IL-1β, NLRP3, and CXCL2 with Escherichia/Shigella and a negative correlation with Eubacterium rectale.*
Zhuang et al. China	2018	43 AD patients and 43 normal controls.	Miseq/16S rRNA V3-V4 regions for fecal samples	1 Phylum level: Bacteroidetes ↑, Actinobacteria ↓ 2 Class level: Actinobacteria and Bacilli ↑; Negativicutes and Bacteroidia ↓ 3 Order level: Lactobacillales ↑; Bacteroidales and Selenomonadales ↓ 4 Family level: Ruminococcaceae, Enterococcaceae and Lactobacillaceae ↑; Lanchnospiraceae, Bacteroidaceae and Veillonellaceae ↓ 5 Genus level: *Bacteroides*, *Ruminococcus*, *Subdoligranulum* ↑; *Lachnoclostridium* ↓
Liu et al. China	2019	97 subjects: 33 AD, 32 aMCI, and 32 healthy controls	Miseq/16S rRNA V3-V4 regions for fecal samples	1 Bacterial diversity decreased in AD patients compared with aMCI patients and healthy controls. 2 Firmicutes ↓ and Proteobacteria ↑ AD compared with healthy controls. 3 More Gammaproteobacteria, Enterobacteriales and Enterobacteriaceae in healthy controls than in aMCI and AD. 4 A significant correlation between the clinical severity scores of AD and the altered microbiomes. 5 PiCRUSt: glycan biosynthesis and metabolism ↑ in AD and aMCI patients, and pathways related to immune system ↓ in AD. 6 Discriminating models can effectively distinguish aMCI and AD from healthy controls, and also AD from aMCI.
Li et al. China	2019	90 subjects: 30 AD, 30 MCI, and 30 healthy controls.	Miseq/16S rRNA V3-V4 regions for fecal samples and blood samples	1 Decreased fecal and blood microbial diversity in AD and MCI. 2 Fecal microbiota in AD: *Dorea*, *Lactobacillus*, *Streptococcus*, *Bifidobacterium*, *Blautia*, and *Escherichia* ↑; *Alistipes*, *Bacteroides*, *Parabacteroides*, *Sutterella*, and *Paraprevotella* ↓. 3 Blood microbiota in AD: *Propionibacterium, Pseudomonas, Glutamicibacter, Escherichia*, and *Acidovora* ↑; *Acinetobacter, Aliihoeflea, Halomonas, Leucobacter, Pannonibacter*, and *Ochrobactrum* ↓. 4 Diagnostic model with fecal key functional bacteria: 93% MCI patients could be identified.
Haran et al. United States	2019	108 elders: 51 had no dementia, 24 AD elders and 33 other dementia types elders	NextSeq 500/Metagenomic Analysis for fecal samples	1 Microbiome diversity differs between AD elders and those with no dementia or other types of dementia. 2 *Bacteroides*, *Alistipes*, *Odoribacter*, and *Barnesiella* ↑; and *Lachnoclostridium* ↓ in AD elders. 3 Accurate classification of AD compared to those without dementia using metagenomic and clinical measures. 4 Accurate classification of AD versus other dementia types using only metagenomic measures. 5 AD microbiome induces lower P-gp expression. 6 Diminished butyrate enzyme-encoding genes in AD.
Guo et al. China	2021	18 newly diagnosed AD, 20 MCI patients, and 18 healthy controls.	Miseq/16S rRNA V3–V4 regions for fecal samples	1 Increased β diversity in patients with AD or MCI 2 *Bacteroides*, *Lachnospira* and *Ruminiclostridium_9* ↓; and *Prevotella* ↑ in AD. 3 *Bacteroides*, *Lachnospira* and *Ruminiclostridium_9* were positively associated with better cognitive functions whereas *Prevotella* was on the contrary. 4 The negative correlation of *Prevotella* with cognitive functions remained among patients with MCI.
Ling et al. China	2021	88 AD patients, and 65 normal controls.	Miseq/ITS2 for fecal samples	1 Fungal diversity unaltered but taxonomic composition altered of in AD. 2 *Candida tropicalis* and *Schizophyllum commune* ↑; *Rhodotorula mucilaginosa* ↓ in AD. 3 *C. tropicalis* and *S. commune* were positively correlated with IP-10 and TNF-α. *C. tropicalis* was negatively correlated with IL-8 and IFN-γ, and *Rhodotorula mucilaginosa* was negatively correlated with TNF-α. 4 PiCRUSt: lipoic acid metabolism, starch and sucrose metabolism ↓in AD.
Ling et al. China	2021	100 AD patients, and 71 normal controls.	Miseq/16S rRNA V3–V4 regions for fecal samples	1 Bacterial diversity↓ in AD. 2 Butyrate-producing genera such as *Faecalibacterium* ↓ in AD, which was positively correlated with MMSE, WAIS, and Barthel scores. 3 Lactate-producing genera such as *Bifidobacterium* ↑ in AD, which was negatively correlated with MMSE, WAIS, and Barthel scores. 4 PiCRUSt: biosynthesis and the metabolism of fatty acids ↓ in AD.

*Aβ, amyloid-β; ACE, abundance-based coverage estimator; AD, Alzheimer’s disease; aMCI, amnestic mild cognitive impairment; CSF, cerebrospinal fluid; CNS, central nervous system; CXCL2, C-X-C motif chemokine ligand 2; IFN-γ, interferon gamma; IL, interleukin; IP-10, interferon gamma-inducible protein 10; MCI, mild cognitive impairment; MMSE, Mini-Mental State Examination; NLRP3, nucleotide-binding domain leucine-rich repeat containing protein 3; PCoA, principal coordinate analysis; PiCRUSt, phylogenetic investigation of bacterial communities by reconstruction of unobserved states; WAIS, Wechsler Adult Intelligence Scale; TNF-α, tumor necrosis factor-alpha.*

Several Chinese AD microbiome studies have also confirmed significant differences in the composition of gut microbiota between healthy individuals and patients with AD, although there were substantial differences across studies. Among the 43 Chinese patients with AD and 43 age- and gender-matched cognitively normal controls from Chongqing (China), Zhuang et al. (2018) found that abundance of Bacteroidetes increased and that of Actinobacteria decreased at the phylum level, while number of *Bacteroides*, *Ruminococcus*, *and Subdoligranulum* increased and that of *Lachnoclostridium* decreased at the genus level in the Chinese AD microbiome. Another study conducted by Liu P. et al. (2019) enrolled 97 participants, including 33 patients with AD, 32 with amnestic mild cognitive impairment (aMCI), and 32 healthy controls from Hangzhou (China) to explore the differences in the microbiome among the three groups. This study characterized gut microbiota in elderly Chinese patients with AD and specifically compared the composition of gut microbiota in the different clinical stages of AD. The AD microbiota in patients of both stages with aMCI and dementia of AD was markedly different from that in HCs, and the altered gut microbiota was significantly correlated with the clinical parameters of AD, especially those indicators representing the severity of AD. Based on the predominant key functional bacteria, they constructed discriminating models that could effectively distinguish aMCI and AD from HC, as well as AD from aMCI. Notably, the abundant family of phylum Proteobacteria, Enterobacteriaceae, was found to be associated with AD, which can help distinguish AD from both aMCI and HC. Similar to Liu’s study, Li B. et al. (2019) also investigated alterations in the gut microbiota and blood microbiota in patients with AD and MCI. They identified differences between AD and normal controls in 11 genera from the feces (increased abundance in AD: *Dorea*, *Lactobacillus*, *Streptococcus*, *Bifidobacterium*, *Blautia*, and *Escherichia*; decreased abundance in AD: *Alistipes*, *Bacteroides*, *Parabacteroides*, *Sutterella*, and *Paraprevotella*) and 11 genera from the blood (increased abundance in AD: *Propionibacterium, Pseudomonas, Glutamicibacter, Escherichia*, and *Acidovora*; decreased abundance in AD: *Acinetobacter, Aliihoeflea, Halomonas, Leucobacter, Pannonibacter*, and *Ochrobactrum*) after adjusting for possible confounding factors (age, sex, BMI, and constipation). Quantitative PCR confirmed increased *Escherichia* and *Lactobacillus* abundance and decreased *Bacteroides* abundance in feces in participants with AD and MCI, while there was a significantly negative relationship between amyloid burden and relative abundance of *Lactobacillus*. However, no differences in genera between AD and MCI were detected, which is inconsistent with Liu’s study. This may imply that alterations in the gut microbiota may occur several years before the onset of dementia, although MCI has a much lower decline in cognitive abilities than AD. They also found that fecal *Akkermansia* is positively correlated with medial temporal atrophy, while higher levels of *Fusicatenibacter*, *Blautia*, and *Dorea* (in the family Lachnospiraceae) were associated with lower MMSE scores and those of *Faecalibacterium, Butyricicoccus*, and *Hungatella* (in the family Clostridiaceae) were related to higher MMSE scores. Gut microbiota in healthy controls does not show AD or MCI-like pattern microbiota, which helps differentiate between normal aging and AD. Using the diagnostic model from fecal samples with all different genera input, 93% (28 of 30) of patients with MCI could be identified correctly. Another Chinese group also determined gut microbiome in patients with MCI or AD. This study excluded the influence of medications or other interventions on AD. Using 16S rRNA sequencing, the changing patterns of gut microbiota were similar in patients newly diagnosed with AD and MCI, with increased β-diversity, decreased *Bacteroides*, *Lachnospira*, and *Ruminiclostridium*_9 abundance, and increased *Prevotella* abundance at the genus level compared with healthy controls (Guo et al., 2021). Inconsistent with the data from the United States cohort, they found that the abundance of potentially protective bacteria such as *Bacteroides* decreased notably in participants with AD and MCI (Cattaneo et al., 2017; Zhuang et al., 2018; Li B. et al., 2019), which could be explained by its ability to protect the intestinal barrier and reverse gut leakiness.

More recently, our group also investigated alterations in the gut microbiota from a relatively larger cohort, including 100 Chinese patients with AD and 71 age- and sex-matched, cognitively normal controls. We found that structural changes in the fecal microbiota were evident in Chinese patients with AD, with decreased alpha-diversity and altered beta-diversity indices (Ling et al., 2021b), which was different from previous studies (Vogt et al., 2017; Zhuang et al., 2018; Liu P. et al., 2019; Guo et al., 2021). This study demonstrated that the abundance of butyrate-producing genera such as *Faecalibacterium* decreased significantly, which was positively correlated with clinical indicators such as the MMSE, WAIS, and Barthel scores in patients with AD. In contrast, abundance of lactate-producing genera, such as *Bifidobacterium*, increased prominently and were inversely correlated with these indicators. As far as the lactate-producing genera were concerned, the traditional probiotics such as *Bifidobacterium* were enriched in patients with AD, which suggested that the genera of *Bifidobacterium* may play vital roles in the pathogenesis of AD. Similar results were also found in patients with *Clostridium difficile* infections (Ling et al., 2014). This indicated that the lactate-producing bacteria, which have been studied extensively in food science and nutrition, are not always beneficial for the human body. Of course, their function should be evaluated cautiously *in vitro* and *in vivo* at the strain level. In addition, as a next-generation probiotic, *Akkermansia* (a typical strain *of A. muciniphila*), plays an important role in metabolic modulation, immune regulation, and gut health protection (Zhai et al., 2019). However, *Akkermansia* in Chinese patients with AD had the strongest negative correlations with clinical indicators such as MMSE, WAIS, and Barthel scores. Previous studies also found that *A. muciniphila* was identified as a key propionate-producing and mucin-degrading organism that is correlated with hippocampal atrophy (Morrison and Preston, 2016; Li B. et al., 2019). Increased levels of *Akkermansia* were observed in gut microbiota of patients with Parkinson’s disease (PD) (Nishiwaki et al., 2020). Based on our observations, *Akkermansia* cannot always be considered a potentially beneficial bacterium; it might be harmful to the gut–brain axis in the context of AD development in the elderly. Similar to the changing patterns of *Lactobacillus* and *Bifidobacterium*, caution should be exercised in future probiotic design and supplementation protocols for patients with AD. In a recent prospective clinical study, patients with AD were administered a mixture of probiotics containing both *Lactobacillus* and *Bifidobacterium* strains. However, probiotic supplementation has an insignificant effect on either cognitive or biochemical indications in patients with severe AD (Agahi et al., 2018). *Faecalibacterium* (typical strain *F. prausnitzii*), a major member of the Firmicutes phylum, is considered to be among the most important bacterial indicators of a healthy gut and can modulate inflammation at the gut epithelium level (Sokol et al., 2008). Decreased *Faecalibacterium* abundance is found in many intestinal disorders and frail (Van Tongeren et al., 2005). A decrease in *Faecalibacterium*, *Coprococcus*, *Blautia*, and *Prevotella* abundance was also observed in PD (Gerhardt and Mohajeri, 2018). Similar to Haran’s findings, the levels of *Faecalibacterium* and other butyrate-producing genera, including *Roseburia*, *Gemmiger*, *Coprococcus*, and *Butyricicoccus*, decreased dramatically in the AD microbiome, which indicated that their metabolites such as butyrate had protective effects in AD development (Haran et al., 2019). Based on the receiving operating characteristic curves, we found that these AD-associated key functional genera such as *Bifidobacterium*, *Faecalibacterium*, *Roseburia*, *Akkermansia*, *Lactobacillus*, and *Enterococcus* can be used as noninvasive biomarkers to discriminate patients with AD from healthy controls. Similar to Li’s findings (Li B. et al., 2019), this study also found that functional dysbiosis, especially the microbial gene functions related to metabolism and biosynthesis of fatty acids, are overactivated in the gut microbiota of patients with AD. Recently, we also explored the alterations in the fungal microbiota in Chinese patients with AD for the first time (Ling et al., 2021a). Fungi are suggested to influence intestinal health and disease by suppressing the outgrowth of potential pathogens, promoting immunoregulatory pathways, and modulating host metabolism (Huseyin et al., 2017; Ni et al., 2017; Sam et al., 2017; Chin et al., 2020). In contrast to previous reports that only focused on gut bacteria, this descriptive study found that the composition of the fungal microbiota was significantly altered. Gut fungal microbiota has been recognized as a novel and important player in the pathophysiology of intestinal and extraintestinal diseases (Huseyin et al., 2017) and is known to have a profound influence on modulating local and peripheral immune responses (Li X. V. et al., 2019). Discordant results in the abovementioned studies, not only the bacterial diversity but also the composition of gut microbiota, may depend on the significant interpersonal variations of gut microbiota. Both physiological and external factors are often unstable over time, influencing gut microbiota. In a two-sample bi-directional Mendelian randomization study, which overcomes the bias owing to confounding and reverse causation, Zhuang et al. (2020) found a protective effect of the host-genetic-driven increase in *Blautia* levels on the risk of AD (per relative abundance: OR, 0.88; 95% CI, 0.79–0.99; *P* = 0.028). Taken together, these cross-sectional clinical studies from different geographical provenances found gut dysbiosis in patients with AD, which indicated a possible involvement of gut microbiota in the development of AD pathology. However, it is still a challenge to establish a causal relationship between the changes in gut microbiota and AD.

Recently, many animal models have been used to explore the causative effects of key gut bacteria in the development of AD. The most commonly used ideal AD model is APP/PS1 transgenic double mice (such as 5 × FAD mice), which express special neurons in the CNS with a chimeric mouse/human amyloid precursor protein (APP) and a mutant human presenilin 1 (PS1). The presence of mutated transgenes (APP and PS1) is the basis for the genetic form of AD in humans (Brandscheid et al., 2017). Previous studies have found that APP/PS1 transgenic mice present detectable gut microbiota alterations after 3 months of age (Brandscheid et al., 2017; Chen Y. et al., 2020), being more pronounced after 6 months of age compared to wild-type animals. Thus, APP/PS1 transgenic mice can be used to evaluate dysbiosis related to memory alterations. Cuervo-Zanatta et al. (2021) found that gut microbiota alterations and cognitive impairment are sexually dissociated in APP/PS1 transgenic mice. Interestingly, alterations in the gut microbiota and its byproducts have been reported in subjects with AD and APP/PS1 transgenic mice in a sex-dependent manner, having a direct impact on brain Aβ pathology in male but not in female mice. There are sex-dependent differences in cognitive skills in wild-type mice, favoring female mice, whereas the cognitive advantage of females was lost in APP/PS1 mice. Sex differences in gut microbiota composition were observed mainly in transgenic mice, with more severe dysbiosis in male than in female mice. Specifically, a decreased abundance of Ruminococcaceae was associated with cognitive deficits in transgenic female mice, while butyrate levels were positively associated with better working- and object recognition memory in wild-type female mice, which implies that sex itself exerted specific influences on the composition of the microbiota in AD pathology. Not only altered in the gut microbiota, Park J. Y. et al. (2017) also demonstrated that serum from APP/PS1 transgenic AD mice demonstrate abundant microbial genomic DNA from gut microbiota derived extracellular vesicle. Without various confounding factors such as human beings, the APP/PS1 mouse models with identical genetic backgrounds and environmental factors can help elucidate the sex-related differences in AD pathology, as the pathological and cognitive differences may be associated only with sex.

Gut microbiota-derived amyloids, similar to CNS amyloids, may contribute to the pathology of progressive neurological diseases with an amyloidogenic component. Similar to patients with AD, Aβ deposits have been observed within the gastrointestinal tract of transgenic mice (Honarpisheh et al., 2020; Sun et al., 2020a). Previous studies have demonstrated that distinct microbial constitutions can influence the development of cerebral amyloidosis (Harach et al., 2017). In germ-free APP transgenic mice, they found a drastic reduction in cerebral Aβ amyloid pathology when compared to control mice with gut microbiota. Both biochemical levels of Aβ and the extent of compact Aβ plaques were consistently decreased in the brains of APP transgenic mice without gut microbiota. After re-colonization of the gut microbiota from conventionally raised APP transgenic mice, but not wild-type mice, the levels of cerebral Aβ increased obviously in the germ-free APP transgenic mice, indicating that alterations in the gut microbiota can directly regulate cerebral Aβ deposition. Gram-negative species such as *Streptomyces, Bacillus, Pseudomonas*, and *Staphylococcus* produce vast quantities of functional amyloids, which can induce cytokine production, inflammation, phagocytosis, and innate immune defense responses that directly impact CNS homoeostasis and drive neuropathology when the gastrointestinal tract epithelial and BBB become significantly more restructured and permeable (Hill and Lukiw, 2015). Zhan et al. (2016) found that lipopolysaccharides (LPS) and *E. coli* fragment K99 colocalize with amyloid plaques in postmortem brain tissue from patients with AD. Higher levels of LPS and *E. coli* fragment K99 can promote myelin aggregates that colocalize with Aβ and resemble amyloid-like plaques. In addition, Javed et al. (2020) found that FapC amyloid fragments (FapCS) of *Pseudomonas aeruginosa* display favorable binding with Aβ and a catalytic capacity to seed peptide amyloidosis, contributing to the aggregation and toxicity of Aβ. The robust seeding capacity for Aβ by FapCS and the biofilm fragments derived from *P. aeruginosa* entailed abnormal behavior pathology and immunohistology and impaired cognitive function in zebrafish. The data revealed a direct cross-seeding linkage between pathogenic bacterial amyloid protein seeds and elevated Aβ fibrillization, neurotoxicity, and AD*-*like pathologies in a zebrafish model for the first time. A previous clinical study also found that several gut species, such as *Bacillus subtilis*, *Escherichia coli*, *Klebsiella pneumonia*, *Mycobacterium* spp., *Salmonella* spp., *Staphylococcus aureus*, *and Streptococcus* spp., are associated with the production of amyloid fibers (Li B. et al., 2019). These amyloid fibers are capable of crossing the intestinal and BBB, and Aβ protein can be deposited in the CNS and promote AD pathogenesis (Larsen et al., 2010; Oli et al., 2012; Pistollato et al., 2016; Paranjapye and Daggett, 2018). Another study in APP/PS1 transgenic mice has found that the gut microbiota alterations with enrichment of inflammation-related bacterial taxa including *Escherichia*-*Shigella*, *Desulfovibrio*, *Akkermansia*, and *Blautia* precede the development of key pathological features of AD, including amyloidosis and plaque-localized neuroinflammation (Chen Y. et al., 2020). Dodiya et al. (2020) also found that synergistic alterations in the gut microbial consortia, rather than individual antimicrobial agents, underlie the observed reductions in brain amyloidosis in APPPS1-21 AD transgenic mice. Sun et al. (2020c) used a novel dementia mouse model to study the influence of gut Aβ on CNS histopathology and function. The researchers observed that enteric Aβ administration produced cognitive impairment and AD-like histopathologies in brain tissue, which provided novel evidence that gut Aβ induces cognitive deficits and AD-related histopathology. Their findings implied that AD may arise in the gut before the brain, opening the possibility of new strategies for the early diagnosis and prevention of AD (Chen Y. et al., 2020; Sun et al., 2020e; Vonderwalde and Finlayson-Trick, 2021). In addition, both *in vitro* (Kumar et al., 2016; Spitzer et al., 2016) and in *vivo* (Kumar et al., 2016) studies have demonstrated that Aβ exerts anti-microbial properties in animal models. A recent study conducted by Dos Santos Guilherme et al. provided the first evidence that acute *in vivo* exposure to Aβ results in a shift in gut microbiota, while chronic exposure to Aβ can trigger an adaptive response of gut microbiota, which could result in dysbiosis in model mice and human patients (Dos Santos Guilherme et al., 2020). Sun et al. (2020c) also demonstrated that Aβ load is likely to occur initially in the gastrointestinal tract and may translocate to the brain, inducing an alteration in gastric function, amyloidosis in the CNS, and AD-like dementia via vagal mechanisms. The cross-talk between gut microbiota and amyloids in AD animal models might provide direct interaction evidence in the pathogenesis of AD targeting the microbiota–gut–brain axis, which may provide new avenues for developing diagnostic biomarkers and therapeutic targets for AD. However, the exact causal molecular mechanisms of gut microbiota in the pathogenesis of AD require further investigation.

## Mechanisms of Gut Microbiota in the Progression of AD

Recent clinical and experimental studies have shown alterations in the gut microbiota in patients with AD. Studies in germ-free animals and animals exposed to pathogenic microbial infections, antibiotic or probiotic intervention, and FMT have suggested a role of gut microbiota in host cognition or AD-related pathogenesis. Despite these growing connections between gut microbiota and brain functions, our understanding of the precise mechanisms underlying these effects is currently lacking. Bidirectional communication along the gut–brain axis links peripheral intestinal function with emotional and cognitive brain centers via neuro-immuno-endocrine mediators, opening a novel avenue to decipher the etiopathogenesis of AD. As mentioned above in the microbiota–gut–brain axis, gut dysbiosis may be involved in the onset and progression of AD through multiple pathological processes resulting from Aβ abnormality, tau phosphorylation, neuroinflammation, neurotransmitter dysregulation, and oxidative stress, which have attracted attention from both gastroenterologists and neurobiologists (Dalile et al., 2019). During dysbiosis, these pathways are dysregulated and associated with altered permeability of the blood-brain barrier and neuroinflammation (Rutsch et al., 2020). The increased permeability of the gut and blood-brain barrier, resulting in large amounts of amyloids and LPS leaking into the circulatory system may contribute to the modulation of signaling pathways and the production of proinflammatory cytokines associated with the pathogenesis of AD. Browne et al. (2013) also reported that dysfunction of the immune system induced by inadequate stimulation of immunity may result in an increased risk of AD through the T cell system. Several studies have found that the functionality of regulatory T (Treg) cells, the fundamental elements of Th1-mediated inflammation, is impaired in patients with AD (Saresella et al., 2010; Pellicanò et al., 2012). Inadequate Treg function in these patients increases the risk of conversion from MCI to AD (Dansokho et al., 2016) while individuals with adequate Treg function may stay longer in the MCI phase (Larbi et al., 2009). In addition, gut dysbiosis-induced peripheral immune responses can propagate bacterial and pro-inflammatory signals to the brain (Matheoud et al., 2019; Madore et al., 2020). Recent microbial endocrinology studies also found that neuroactive molecules such as neurotransmitters produced by gut microbes directly contribute to the communication between the gut and the brain (Yano et al., 2015; Pokusaeva et al., 2017; Sun et al., 2020a). These neurotransmitters including 5-hydroxytryptophan (5-HT), dopamine, acetylcholine, γ-aminobutyric acid (GABA), and serotonin, produced by bacteria belonging to *Lactobacillus, Bifidobacterium*, *Enterococcus*, and *Streptococcus* species, can influence brain cell physiology directly and indirectly. In addition, gut microbial-origin gasotransmitters, including SCFAs and gaseous substances, such as nitric oxide (NO), carbon monoxide (CO), hydrogen sulfide (H_2_S), hydrogen, methane, and ammonia, perform important functions in neurophysiological, biochemical, microbiological, and medical terms, which may be involved in the pathogenesis of AD (Szabo, 2010; Oleskin and Shenderov, 2016, 2019; Sun et al., 2020d).

Mounting evidence has demonstrated that the gut microbiota interacts with AD pathogenesis via various pathways, suggesting that the gut microbiota has gone from being the forgotten organ to a potential key player in AD pathology (Seo and Holtzman, 2020). We summarized the underlying mechanisms of gut microbiota in the regulation of brain function in AD. These mechanisms, alone or in combination, actively participate in the development of AD.

### Aβ and tau Phosphorylation Pathways

Gut microbiota can affect the deposition of Aβ proteins in the brain in many ways. Initiation of Aβ in the brain is elusive; however, different *in vitro* and *in vivo* studies have claimed that amyloids produced by GM may cross-seed Aβ deposition (Friedland et al., 2020). An *in vitro* study found that soluble *E. coli*-derived LPS accelerated the polymerization of Aβ monomers into insoluble aggregates (Pistollato et al., 2016). The structure and immunogenicity of amyloids secreted by *Streptomyces, Staphylococcus, Pseudomonas, Bacillus*, and *E. coli*, are similar to those of Aβ42, and can bind to TLR2 receptors on microglia, activating these cells to release inflammatory factors, and promoting inflammatory responses. These strains produce curli, TasA, CsgA, FapC, phenol soluble modulins, etc., amyloids that promote the misfolding of Aβ fibrils and oligomers (Shabbir et al., 2021). The microglia monitoring system can identify Aβ42 peptides and their misfolded aggregates. The deposition of Aβ oligomers and fibrils formed by misfolding of proteins may lead to pathological changes in characteristics of AD. The inability of microglial cells to process these toxic and proinflammatory inclusions is thought to be the molecular basis of elevated oxidative stress, abnormal immune activation, and chronic diseases. In addition, amyloids derived from gut microbiota induce the release of proinflammatory factors such as interleukin (IL)-17A and IL-22. These two pro-inflammatory factors are directly related to AD and may enter the brain through the gastrointestinal tract and the blood-brain barrier. IL-17-expressing Th cells migrate to the CNS throughout the gut-associated lymphoid tissue, and this is essential for neurodegeneration because of their interactions with microglia, which can contribute to the clearance of Aβ molecules and tau aggregation (Janeiro et al., 2021). They further trigger immune activity, the release of reactive oxygen species (ROS), and activation of the TLR2/1, CD14 and nuclear factor kappa B (NF-κB) signaling pathways that participate in neurodegenerative diseases (Pistollato et al., 2016). Aβ is cleared by the liver, and gut microbiota disorders affect the clearance of Aβ by affecting the gut mucosal barrier and energy homeostasis (Hrncir et al., 2019). Recently, Chen C. et al. (2020) found that gut dysbiosis contributes to amyloid pathology, associated with CCAAT/enhancer binding protein β/asparagine endopeptidase (C/EBPβ/AEP) signaling activation in AD mouse models. Prebiotic R13, which induces *L. salivarius* growth, antagonizes the C/EBPβ/AEP axis, suppresses amyloid aggregates in the gut and mitigates gut leakage and oxidative stress. Aβ proteotoxicity has been implicated in intracellular neurofibrillary tangles composed of hyperphosphorylated tau protein, which are also characteristic of AD pathology. The microtubule-associated protein tau is abnormally phosphorylated and forms aggregates of paired helical filaments in AD and other tauopathies. p-tau is a marker of tau phosphorylation and is believed to be associated with neurofibrillary tangle pathology, with higher levels reflecting a more intense tau phosphorylation process. Vogt et al. (2018) found that the gut microbiota-derived metabolite trimethylamine N-oxide (TMAO) levels are elevated in the CSF of individuals with MCI and AD dementia, and that CSF TMAO levels are associated with tau pathology. Wang et al. (2015) demonstrated that exposure to *Helicobacter pylori* filtrate induces Alzheimer-like tau hyperphosphorylation by activating glycogen synthase kinase-3β (GSK-3β). Sun et al. (2019a) also found that decrease in abundance of gut bacteria such as *Marvinbryantia*, *Lactobacillus, Streptococcus, unclassified_Erysipelotrichaceae*, and *Eubacterium_brachy_group* were negatively correlated with tau pathology in the brain in a tauopathy model, suggesting that the decrease in these microbiota may be closely related to the severity of tauopathies. Recently, Wei et al. (2020) found that outer membrane vesicles, produced by the gut microbiota, increase the permeability of the BBB and promote the activation of astrocytes and microglia, inducing an inflammatory response and tau hyperphosphorylation by activating the GSK-3β pathway, ultimately leading to cognitive impairment. Using a recently developed AD-like pathology with amyloid and neurofibrillary tangles (ADLP^APT^) transgenic mouse model of AD, Kim et al. (2020) found that long-term, frequent transfer and transplantation of fecal microbiota of healthy wild-type mice alleviates Aβ deposition, tau pathology, reactive gliosis and memory impairment. Although the comprehensive pathogenesis of tauopathies remains unclear, these studies indicate that modulation of the gut microbiota may be a potential strategy for tauopathy treatment for AD. Understanding these roles and mechanisms of gut microbiota underlying the two key pathological components of AD pathogenesis can help develop promising anti-amyloid aggregation and anti-tau phosphorylation therapies in AD with specific gut microbes.

### Inflammation

Inflammation appears to occur in all neurodegenerative disorders such as AD and PD and distinct immune factors are associated with each type of disorder. Neuroinflammation is a physiological response to exogenous and endogenous insults that target the CNS and represents a protective response in the brain, but excessive inflammatory responses are detrimental to the CNS. Several immune pathways are involved in CNS homeostasis and AD-associated neuroinflammation. At the molecular level, the interaction between the brain and surrounding environment plays a vital role in the occurrence and development of brain diseases (Jiang et al., 2015). Germ-free animals, and specific pathogen-free animals treated with antibiotics, which demonstrated the extreme gut dysbiosis or gut decontamination, showed impaired microglial maturation and immune response to bacterial stimuli. Recently, several studies have shown that gut dysbiosis is involved in the pathogenesis and development of AD by affecting the immune system and inflammatory responses (Qureshi and Mehler, 2013; Wang et al., 2016; Sun et al., 2020a). The higher abundance of pro-inflammatory gut microbiota is accompanied by enhanced systemic inflammation and neuroinflammatory processes. Gut dysbiosis leads to defects in the maturation, differentiation, and function of microglia, the activation of which contributes to the progression of AD. Several gut-specific microorganisms produce nitric oxide and APP and activate microglia, thereby aggravating the development of AD (Sun et al., 2020d). Changes in microbial composition can trigger peripheral immune responses by activating immune components and regulating the levels of pro-inflammatory cytokines in the brain (Harach et al., 2017; Park A.-M. et al., 2017). A recently discovered CD4^+^ T cell subset, called Th17 cells, found on the mucosal surface of the lung, skin, and intestine, plays important roles in autoimmunity and immunity (Weaver et al., 2013; Eyerich et al., 2017). Th17 cells activate microglia in the CNS, leading to local production of IL-1β, tumor necrosis factor-α (TNF-α), and IL-6, which strongly suggests its key role in neuroinflammation. CD4^+^ Th17 cells are not found in the germ-free mouse intestine, indicating that this subset is generated in response to the microbiota (Weaver et al., 2013). *Candidatus arthromitus*, more popularly known as segmented filamentous bacteria, is a potent bacterial inducer of Th17 cells in the intestine (Ivanov et al., 2009). With specific anti-inflammatory bacteria or transfer of healthy fecal microbiota, neuroinflammation can be modulated, and AD pathology and symptoms can be improved.

Several inflammation-inducing stimuli, such as damage-associated molecular patterns (DAMPs) and pathogen-associated molecular patterns (PAMPs), are recognized by multiprotein complexes called inflammasomes (Piancone et al., 2021). These DAMPs and PAMPs can activate pattern recognition receptors (PRRs) expressed on glial cells to respond to brain injury or pathogen invasion. PRRs, including Toll-like receptors (TLRs), the nucleotide-binding oligomerization domain leucine rich repeat-containing receptors (NLRs), are important innate immune proteins expressed on the surface and within the cytoplasm of a multitude of cells, both immune and otherwise, including epithelial, endothelial, and neuronal cells. Recognition of commensal bacteria by PRRs is critical for maintaining host-microbe interactions and homeostasis, including behavior via the gut–brain axis. The impaired gut mucosal barrier and BBB increase bacterial LPS levels, which can induce inflammation and disrupt the BBB function (Lin et al., 2020). LPS can trigger TLR4 pro-inflammatory cascades in monocytes, Kupffer cells, and macrophages, resulting in the activation of downstream signaling pathways, including factor kappa β (NF-kB) and mitogen-activated protein kinase, leading to inflammation driven by TNF-α and IL-6 (Morrison and Preston, 2016). NF-κB activity and TNF-α and cyclooxygenase-2 levels are upregulated in a transgenic AD mouse model (5 × FAD-Tg) (Lee et al., 2019). LPS-induced neuroinflammation has been shown to induce Aβ aggregation and memory impairment, as well as astrocyte activation, with a significant reduction in the expression levels of brain-derived neurotrophic factor (BDNF), while enhancing the expression of brain parenchymal cell apoptosis markers such as cytochrome C, Bax, caspase-9, and caspase-3 (Song et al., 2013; Calabrese et al., 2014; Badshah et al., 2016). Some microbiota had an anti-inflammatory activity identified on the basis of human clinical data, including *F. prausnitzii* (Sokol et al., 2008; Wang et al., 2014), *E. rectale*, *B. longum* (NK46), and *B. fragilis* (Lee et al., 2019; Markowiak-Kopeć and Śliżewska, 2020; Sun et al., 2020d; Zhang M. et al., 2020). Lee et al. found that *B. longum* (NK46) was orally administered to 5 × FAD mice and induced anti-inflammatory effects (decrease in LPS levels, NF-κB activation, and TNF-α expression), changes in the intestinal microbiota composition of the recipients (increase in Bacteroides abundance and reduction in abundance of Firmicutes and Proteobacteria phyla), and suppression of Aβ accumulation in the hippocampus (Lee et al., 2019). Recently, inflammasome complexes assemble upon cell activation because of exposure to microbes, danger signals, or stress, leading to the production of pro-inflammatory cytokines (IL-1β and IL-18) and pyroptosis, which lead to neurodegeneration. Evidence suggests that there is a reciprocal influence of microbiota and inflammasome activation in the brain. As mentioned by Feng et al., the NLRP3 inflammasome is the most characterized in neurodegenerative diseases, especially in AD, which acts as a key player in coordinating the host physiology and shaping the peripheral and central immune/inflammatory responses in CNS diseases (Feng et al., 2020; Pellegrini et al., 2020). Heneka et al. (2013) found overexpression of the NLRP3 inflammasome in the postmortem brains of human patients with AD, which is associated with inflammatory crystals and aggregated Aβ since the earliest stage of AD (Song et al., 2017; Venegas et al., 2017). Recently, there has been pioneering evidence supporting the existence of a microbiota–gut–inflammasome–brain axis, in which enteric bacteria modulate, via NLRP3 signaling, inflammatory pathways that, in turn, contribute to brain homeostasis (Rogers et al., 2016). Besides caspase-1-dependent NLRP3 activation, Pellegrini et al. have described a non-canonical activation, depending on caspase-11 in mice (caspases 4 and 5 in humans). Gram-negative bacteria (i.e., *Citrobacter rodentium*, *Escherichia coli*, *Legionella pneumophila*, *Salmonella typhimurium*, and *Vibrio cholerae*) can regulate non-canonical NLRP3 activation via activation of TLR4-MyD88 and toll/IL-1 receptor homology-domain-containing adapter-inducing interferon-β (TRIF) pathways, with consequent transcription of IL-1β, IL-18, NLRP3, interferon regulatory factor (IRF)-3, and IRF7 genes through NF-κB activation (Pellegrini et al., 2017). Lowe et al. (2018) showed that the depletion of gut microbiota with an antibiotic cocktail influenced inflammasome signaling in the intestine, blood and brain. Cohousing wild-type and NLRP3^–/–^ animals reshaped the gut microbiota and prevented the effects of NLRP3 inflammasome gene depletion on locomotion and behavior (Zhang et al., 2019). More recently, Shen et al. (2020) showed that FMT from patients with AD to APP/PS1 transgenic mice is associated with an increased expression of intestinal and circulating NLRP3 inflammasome components, including NLRP3, caspase-1 and IL-1β, as compared with APP/PS1 subjected to FMT from healthy subjects, which displayed a more severe impairment of cognitive functions and central neurogenic/inflammatory responses. Restoration of the gut microbiota can inhibit central inflammation caused by NLRP3. Of course, it is difficult to establish a clear relationship between altered gut microbiota, activation of NLRP3 inflammasome signaling, and brain pathology. Future studies are needed to elucidate the molecular and cellular mechanisms underlying the interactions between gut microbiota and NLRP3 inflammasome, as well as their role in the regulation of the gut–brain axis in both physiological and pathological conditions. Taken together, gut microbiota dysbiosis promotes neuroinflammation or activates the inflammasome signaling pathways, thereby contributing to brain disorders, suggesting that gut microbiota can be used as a promising therapeutic strategy to inhibit inflammation in AD.

### Metabolic Dysfunctions

The core functions of gut microbiota are the production of various bioactive metabolites that affect host health and disease (Clarke et al., 2019; Mccarville et al., 2020). These microbiota-derived bioactive metabolites such as SCFAs and neurotransmitters including acetylcholine, GABA, and serotonin can modulate many immune system pathways that in turn influence behavior, memory, learning, locomotion, and neurodegenerative disorders (Rutsch et al., 2020). Microbial metabolites can communicate through dynamic bidirectional pathways within the microbiota–gut–brain axis to mediate host brain immunity and physiology (Mccarville et al., 2020). These metabolites exert direct effects after being transported across the BBB or indirectly through immune, neuroendocrine, or vagal mechanisms (Fulling et al., 2019; Mccarville et al., 2020). Sun et al. (2020d) have demonstrated that metabolites from the gut microbiota and its components can modulate immune cells, resident immune cells (such as microglia) of the brain, and infiltrating peripheral T cells through inflammatory pathways. Immune cells that reside in the brain are key players in learning and cognitive functions. Microglia are highly dynamic monitoring agents of the immune system that combat systemic infections (Silver and Curley, 2013). The enteric nervous system interacts with the autonomic nervous system and the CNS via neurotransmitters (adrenaline, noradrenaline, and acetylcholine), as well as sensory and motor neurons, all of which convey signals from the gut to the brain. A study showed that the gut microbiota can convert glutamate (an excitatory transmitter) into GABA, which participates critically in human brain development, particularly emotional and behavioral development (Zhang M. et al., 2020). Genome-based metabolic modeling of the human gut microbiota revealed that several genera have the ability to produce microbial neurotransmitters such as GABA and serotonin, which have been consistently shown to play a key role in the regulation of brain function. A meta-analysis of 35 observational studies reported that increased GABA levels were associated with a lower risk of AD (Manyevitch et al., 2018). Wu et al. (2021) also found that gut microbiota metabolites such as tryptophan metabolites, SCFAs and lithocholic acid are different between AD and controls, which are correlated with altered microbiota and cognitive impairment. In addition, urine and serum serotonin concentrations were found to be significantly lower in patients with AD than in controls (Whiley et al., 2021). Metabolic dysfunction is a basic characteristic of AD. However, most gut microbiota-associated metabolic pathways have been predicted based on sequencing microbiome data (Ling et al., 2021a,b). Technical sequencing and bioinformatic limitations hinder the discovery of specific biomarkers of microbes or metabolites conserved across studies.

Short-chain fatty acids with five or fewer carbons that are available to the gut microbiota and are primary end-products of fermentation of non-digestible carbohydrates can regulate the CNS physiology and behavior of the host (Samuel et al., 2008; Morrison and Preston, 2016; Zhang M. et al., 2020). The diversity of the gut microbiota determines the proportion of each SCFA produced. Acetate is produced by a variety of microbes such as *Lactobacillus* and *Bifidobacterium*, whereas propionate is produced mainly by *A. muciniphila*, and butyrate is produced by *F. prausnitzii*, *Ruminococcus bromii*, *E. rectale*, and *E. hallii* (Morrison and Preston, 2016). Butyrate, acetate, and propionate promote the proliferation of regulatory T cells, offer energy for host cells, and accommodate enteroendocrine cells (Zmora et al., 2017; Gribble and Reimann, 2019; Li J. M. et al., 2019). A previous animal study found decreased levels of SCFAs in AD (Zhang et al., 2017). SCFAs can affect the brain and behavior via various molecular mechanisms, such as inhibition of histone deacetylase (HDAC), induction of enteroendocrine signaling, vagus nerve activation, and anti-inflammatory properties (Cryan et al., 2019). These actions of SCFAs are mediated through activation of the G-protein-coupled receptor (GPR) 41 and GPR43. SCFAs are known to beneficially modulate the peripheral and central nervous systems and have been suggested to play a central role in AD. They can interfere with various Aβ peptides, thereby potently inhibiting Aβ fibril aggregation *in vitro* (Ho et al., 2018), possibly reducing the accumulation of neurotoxic oligomers in the brain. Marizzoni et al. (2020) reported a novel association between gut microbiota-derived SCFAs and systemic inflammation with brain amyloidosis via endothelial dysfunction in a clinical study. Erny et al. (2015) also suggested that SCFAs may help modulate the maturation and function of microglia in the brain. Recently, increasing attention has been paid to butyrate for its protective role against the pathophysiological processes of AD. Our clinical study suggested decreased butyrate-producing genera in patients with AD (Ling et al., 2021b). Butyrate is a multifunctional molecule that exerts beneficial neuroprotective effects and improves brain health. Butyrate can also interfere with Aβ1-40 oligomerization and exert multiple effects against neuropsychiatric disorders as an inhibitor of HDAC, which can improve cognitive memory performance in a 5 × FAD mouse at an early disease stage (Griseri et al., 2003; Ho et al., 2018; Fernando et al., 2020). Sun et al. (2020b) found that butyrate protects N2a cells from Aβ-induced cell damage by activating GPR109A *in vitro*. Our previous studies also demonstrated that butyrate could exert neuroprotective effects on depression, PD, AD and traumatic brain injury in model mice (Liu et al., 2015; Sun et al., 2015, 2016a,b, 2020a; Li et al., 2016). With regard to AD, butyrate can improve the gut and brain-blood barriers, attenuate microglia-mediated neuroinflammation, and influence glucose metabolism in the hippocampus by regulating the microbiota–gut–brain axis. Butyrate can be considered a pathophysiological link between the gut microbiota and AD pathology.

Gut microbiota-derived neurotransmitters, such as acetylcholine, GABA, dopamine, and serotonin, play a key role in modulating the gut–brain axis, while their abnormal alterations are involved in the pathogenesis of AD. Deficiency of the cholinergic system in the brain is the neurotransmitter most closely associated with AD (Stanciu et al., 2019). In addition, the noradrenergic locus coeruleus and tuberomammillary nucleus, which secrete norepinephrine and histamine, respectively, exhibit a distinct neuronal loss in the brain with AD (Oh et al., 2019). These neurotransmitters regulate brain function through blood circulation or nerve conduction. GABA, the major inhibitory transmitter in the adult mammalian brain, has been reported in several species belonging to the families Bifidobacteriaceae, Lactobacillaceae, Bacteroidaceae, Enterococcaceae, Propionibacteriaceae, and Streptococcaceae (Duranti et al., 2020; Altaib et al., 2021). *Bifidobacterium* abundance such as *B. adolescentis* is associated with high fecal GABA content in healthy human subjects (Duranti et al., 2020; Altaib et al., 2021). Studies in the postmortem brains of patients with AD and animal models have shown substantially reduced levels of GABA in the subregions of the temporal cortex. GABAergic dysfunctions, including reduction of GABA receptors, loss of GABAergic neurons and synapses, aberrant GABA production in reactive astrocytes, and imbalance between excitatory and inhibitory signals of the CNS, are early events occurring in the brains of patients with AD and animal models (Govindpani et al., 2020; Zheng et al., 2021). Acetylcholine, produced by *Bacillus subtilis* and *Lactobacillus plantarum*, can reduce the production of IL-6, TNF-α, and IL-1β through the COX-2 pathway in astrocytes, inhibit pro-inflammatory cytokines by upregulating JAK2/STAT3 and PI3K/AKT signaling pathways in microglia and regulating immune homeostasis (Velazquez et al., 2019; Qian et al., 2021). Dopamine can be produced by *Bacillus* and *Escherichia* in humans. Activation of the dopamine receptor D1 (DRD1) accelerates the degradation of the NLRP3 inflammasome via cyclic adenosine monophosphate, while DRD2 and DRD3 in astrocytes perform anti-inflammatory and pro-inflammatory functions, respectively (Montoya et al., 2019; Qian et al., 2021). 5-HT is an important neurotransmitter synthesized from tryptophan metabolism in enterochromaffin cells (Kennedy et al., 2017), which can be produced by *Corynebacterium* spp., *Streptococcus* spp., and *E. coli*. In addition, 5-HT biosynthesis can be regulated by SCFAs such as acetate and butyrate, which can have a significant impact on homeostasis, especially on gut motility and platelet function (Yano et al., 2015). A recent study also found that high dietary fiber intake upregulates the expression of 5-HT and suppresses neuroinflammation (Liu Z. et al., 2020). Taken together, these bacterial metabolites and neurotransmitters can participate in regulating brain functions directly or indirectly and play vital roles in the development of AD.

### Oxidative Stress

Oxidative stress plays an important role in AD pathology. Pro-inflammatory cytokines and some metabolites of the kynurenine pathway trigger powerful bursts of ROS, thereby damaging neurons and glial cells. Gut microbiota may influence the levels of oxidative stress in the CNS, either by increasing the oxidant components or by interfering with antioxidant systems. *Lactobacillus*, *E. coli*, and *Bifidobacterium* can convert nitrate and nitrite into nitric oxide, the elevations of which have been shown to increase the permeability of the BBB and react with superoxide to form peroxynitrite, a potent oxidizing agent contributing to neurotoxicity in AD. The levels of oxidative markers are directly proportional to the degree of cognitive impairment and brain weight. A mutual relationship between oxidative stress and Aβ production and aggregation in AD has been identified. Oxidative stress enhances Aβ deposition, whereas Aβ triggers oxidative reactions. Researchers have proposed a mechanism by which T cell-mediated inflammation is enhanced after oxidative stress (Dumitrescu et al., 2018). In addition, gut pathogenic bacteria such as *Salmonella* and *E. coli* are able to degrade sulfur amino acids, leading to hydrogen sulfide production in the gut, which leads to a decreased mitochondrial oxygen consumption and overexpression of proinflammatory mediator genes such as IL-6 (Beaumont et al., 2016). Hydrogen is a highly diffusible bioactive gas produced mainly by anaerobic cocci, strains of the genus *Clostridium*, and members of the *Enterobacteriaceae* family. Owing to its anti-apoptotic, anti-inflammatory, and antioxidant properties, hydrogen has been considered as a preventive and therapeutic medical gas (Ohta, 2014). Reduced availability of hydrogen has been observed in neurodegenerative diseases. Gut dysbiosis may lead to reduced hydrogen production and limit gas supply to neurons in the CNS. In contrast, the accumulation of hydrogen in the gastrointestinal tract has an inhibitory effect on the fermentation of polysaccharides, resulting in a reduction in its products, including SCFAs. These findings suggest that the symbiotic relationship between hydrogen-producing bacteria and hydrogenotrophic microbes, such as *Methanobrevibacter smithii*, is essential for homeostasis and redox state. Methane also acts as an antioxidant, anti-inflammatory, and anti-apoptotic gas (Huai et al., 2014). It protects against oxidative stress by increasing the levels of superoxide dismutase and reducing the levels of malondialdehyde and 3-nitrotyrosine (Huai et al., 2014). Taken together, alterations in the gut microbiota favor oxidative stress and affect the immunological and inflammatory status of the host, which can influence the pathogenesis of AD.

## Gut Microbiota Modulation for AD Treatment

Considering the importance of gut microbiota in patients with AD, it is apparent that the maintenance of a healthy microbiota is fundamental for an individual’s well-being. Generally, gut microbiota can be modulated via dietary intervention, microecological regulators (psychobiotics), and FMT. The modulation of gut homeostasis triggers multiple mechanisms, including anti-inflammatory and antioxidant effects, upregulation of neuroprotective hormones, restoration of impaired proteolytic pathways, amelioration of energy homeostasis with consequent decrease of AD molecular hallmarks, and improvement of behavioral and cognitive performances (Bonfili et al., 2020).

### Diet-Based Microbiota Modulation

The interplay between diet, gut microbiota, and the host is a major factor impacting health. Diet is one of the major factors involved in shaping gut microbiota composition across the lifespan. Dietary habits have been recognized as one of the drivers of microbial composition and diversity, and the impact of both individual nutrients and dietary patterns on the microbiota have been extensively explored (Thelen and Brown-Borg, 2020; Berding et al., 2021). Numerous studies have suggested a link between dietary patterns and AD incidence, presenting diet as a modifiable risk factor (Hu et al., 2020; Berding et al., 2021). Poor dietary habits and aging, along with inflammatory responses because of dysbiosis, may contribute to the pathogenesis of AD. The Mediterranean diet (MedDiet), considered an anti-inflammatory diet, has been shown to reduce the occurrence of several chronic diseases. Valls-Pedret et al. (2015) demonstrated that the MedDiet is associated with preserved cognitive function in elderly adults. Epidemiological findings are consistent in showing that adherence to a Mediterranean diet characterized by high intake of fruit, vegetables, cereals, and legumes, and low intake of meat, high-fat dairy, and sweets, is consistently associated with a lower risk of AD (Yusufov et al., 2017). In a Mediterranean study involving over 16,000 middle-aged and elderly participants followed up for over 20 years, Andreu-Reinón et al. (2021) found that adherence to the MedDiet was associated with a 20% lower risk of AD overall in the EPIC-Spain Dementia Cohort. Adherence to the MedDiet increases the diversity of microbiota after 1 year of intervention, with higher ratio of Firmicutes/Bacteroidetes and higher levels of SCFAs, which is negatively associated with inflammatory markers. A recent study conducted by Ghosh et al. (2020) found that MedDiet is effective in altering gut microbiota, reducing frailty, and improving cognitive function. These microbiota changes are primarily driven by an increase in the intake of fibers, vitamins (C, B6, B9, thiamine), and minerals (Cu, K, Fe, Mn, and Mg) in the MedDiet intervention group, while changes in the controls were associated with an increase in fat intake (saturated fats and mono-unsaturated fatty acids). MedDiet has been proposed as one of the top five modifiable factors against AD and cognitive decline.

The ketogenic diet (KD) is a very low-carbohydrate, adequate protein, and high-fat diet that mimics the fasting state (Dewsbury et al., 2021). Intermittent fasting (IF) is the lifestyle of eating within a specific period and fasting for the rest of the day. Both measures promote fat utilization in the body by producing ketone bodies, which are products of the incomplete oxidization of fatty acids. Yin et al. (2016) reported the protective mechanism of ketones in an AD mouse model, including blockage of Aβ42 entry into neurons, reduced oxidative stress, and improved synaptic plasticity. Recent studies have shown the role of KD in remodeling the composition of the gut microbiome, thereby facilitating protective effects in various CNS disorders including AD. KD can improve neuropathological and biochemical changes associated with AD and preserve cognition in patients with mild AD (Carranza-Naval et al., 2021). A modified Mediterranean-KD can modulate gut microbiome and metabolites (mainly SCFAs) in association with improved AD biomarkers in CSF (Nagpal et al., 2019). Ma et al. (2018) found that KD started in the early stage can enhance brain vascular function, increase beneficial gut microbiota, improve metabolic profile, and reduce the risk of AD. In addition, IF has been proposed to produce neuroprotective effects by reducing insulin resistance, improving metabolic regulation, increasing autophagy, reducing inflammation and neuroinflammation, and increasing BDNF level (Park et al., 2020). IF can promote adult hippocampal neuronal differentiation by activating GSK-3β in 3 × Tg-AD mice, leading to the inhibition of insulin and protein kinase A signaling pathways and activation of adenosine monophosphate-activated protein kinase and BDNF pathways. Likewise, studies in elderly patients have shown the neuroprotective effects of caloric restriction on memory (Witte et al., 2009). In summary, dietary interventions are generally safer and more advantageous than drug-based therapies because they are cheap and easy to handle, thus reducing the burden on caregivers of patients with AD.

### Probiotics

As an effective strategy to modulate gut microbiota, probiotics have been used to restore gut microbiota to a healthy status and maintain gut homeostasis, thereby improving human health and preventing diseases. According to the definition of the WHO in 2002, underscored by the International Scientific Association for Probiotics and Prebiotics in 2013, probiotics are live microorganisms that, when administered in adequate amounts, confer health benefits to the host (Hill et al., 2014). Probiotics have recently attracted attention in brain function and health because they improve gut microbiota by positively affecting the gut–brain axis. These probiotics attempt to correct imbalances in the microbiota–gut–brain axis are referred to as psychobiotics, which can release neuroactive substances directly influencing the human brain, psyche, and behavior (Sarkar et al., 2016). Animal studies indicate that probiotic supplementation can restore gut microbiota, improve the integrity of the gut barrier and BBB, reduce neuroinflammation, improve oxidative stress, reduce cognitive decline, and reduce insulin resistance (Kobayashi et al., 2017; Athari Nik Azm et al., 2018; Tamtaji et al., 2019). The most commonly used probiotics belong to the genera *Lactobacillus* and *Bifidobacterium*. Shamsipour et al. (2021) found that *B. bifidum* combined with *L. plantarum* with exercise training can alleviate neurotoxicity of Aβ and spatial learning via acetylcholine in an AD rat model. Liu M. et al. (2020) found that *L. reuteri* SL001 plays a positive role in adjusting the gut microbiota structure in AD model mice. In male 5 × FAD-transgenic (6 months old) and aged (18 months old) mice, oral administration of *B. longum* NK46 can suppress gut dysbiosis and LPS production, resulting in the attenuation of cognitive decline with the regulation of neuroinflammation via the microbiota–gut–brain axis (Lee et al., 2019). Recently, Yang et al. (2020) found that ProBiotic-4 (including *B. lactis*, *L. casei*, *B. bifidum*, and *L. acidophilus*) can modulate gut microbiota dysbiosis and microbiota–gut–brain axis deficits in AD mice via inhibition of both TLR4- and RIG-I-mediated NF-κB signaling pathway and inflammatory responses, resulting in improvement of cognitive dysfunction. In addition, 12-weeks of treatment with memantine plus *L. plantarum* ameliorated cognitive deterioration, decreased Aβ levels in the hippocampus, protected neuronal integrity and plasticity, and reduced TMAO synthesis and neuroinflammation (Wang Q. J. et al., 2020). In addition to *Lactobacillus* and *Bifidobacterium*, our previous study demonstrated that *Clostridium butyricum* can prevent cognitive impairment, Aβ deposits, microglial activation, and production of TNF-α and IL-1β by regulating the microbiota–gut–brain axis, which is mediated by the metabolite butyrate (Sun et al., 2020a). Butyrate is beneficial for proper vagal afferent signals in patients with AD and participates in learning and memory, regulating neuronal growth and survival as well as synaptic differentiation via intracellular Ca^2+^ signaling and by maintaining BDNF levels (Barichello et al., 2015; Goswami et al., 2018). *Agathobaculum butyriciproducens* SR79, a strict anaerobic and butyrate-producing bacterium, can improve cognitive impairment by decreasing microglial activation and reducing Aβ plaque deposition in APP/PS1 mice (Go et al., 2021). To date, most of the existing evidence has been based on *in vitro* and *in vivo* experimental studies (Wang F. et al., 2020). Recently, a randomized, double-blind, placebo-controlled, multicenter trial in Korea found that *B. bifidum* BGN4 and *B. longum* BORI supplementation is beneficial for improving cognitive and mental health in community-dwelling healthy older adults with changes in gut microbial composition (Kim et al., 2021). Another clinical trial showed that *B. breve* A1 supplementation may have beneficial effects on cognitive function in elderly Japanese subjects with memory complaints (Kobayashi et al., 2019). In three clinical randomized controlled trials, clinically confirmed patients with AD treated with probiotics (mainly *Lactobacillus* and *Bifidobacterium* strains) for 12 weeks showed no beneficial effect on cognitive function (Akbari et al., 2016; Krüger et al., 2021). This may be due to insufficient intervention period, different disease severity, and changes in dietary patterns. However, Tamtaji et al. (2019) found that probiotics (including *L. acidophilus*, *B. bifidum*, and *B. longum*) and selenium co-supplementation for 12 weeks in patients with AD improved cognitive function and some metabolic profiles. These facts reveal that psychobiotics have demonstrated significant potential in the deceleration of AD progression when consumed as a lone or combination supplement, whereas the appropriate strains, doses, time of treatment, routes of administration, and safe use of probiotics for AD need to be studied in the future.

### Prebiotics

Prebiotics are short-chain carbohydrates that alter the composition and metabolism of gut microbiota in a beneficial manner. Prebiotics can act as specific fermentation substrates for SCFA-producing probiotic genera such as *Bifidobacteria* and *Lactobacilli*, leading to an increase in anti-inflammatory metabolites. Recent studies in animal models and healthy volunteers have evaluated the effects of prebiotics on psychiatric symptoms. Sun et al. (2019b) found that fructooligosaccharides can reverse the altered microbial composition, ameliorate cognitive deficits and pathological changes, upregulate the expression levels of synapsin I, and decrease phosphorylated levels of JNK in APP/PS1 transgenic mice by regulating the gut microbiota-GLP-1/GLP-1R pathway. Lactulose, the first commercially available prebiotic and commonly used as a food additive, can attenuate short-term memory and learning retrieval in AD mice. Recently, the therapeutic mechanism of lactulose in AD has been uncovered. Lee et al. (2021) found that pretreatment with lactulose can modulate the gut microbiome and insulin sensitivity, decrease neuroinflammation, and increase the levels of autophagy pathways. These findings suggest that the neuroprotective effect of lactulose is partially caused by autophagy activity. Another prebiotic, sodium oligomannate (GV-971), received its first approval in November 2019 in China for the treatment of mild to moderate AD to improve cognitive function. GV-971 is an orally administered mixture of acidic linear oligosaccharides derived from marine brown algae. GV-971 can reconstitute the dysbiosis of gut microbiota, reduce metabolite-driven peripheral infiltration of immune cells into the brain, inhibit neuroinflammation, and reverse cognitive impairment (Wang et al., 2019). In addition, GV-971 can easily penetrate the BBB to directly bind to Aβ and inhibit Aβ fibril formation (Wang et al., 2007). Synbiotics (a combination of probiotics and prebiotics) and postbiotics (functional bioactive compounds such as SCFAs) can also be used to treat AD by modulating the gut microbiota. Taken together, these studies suggest that personalized prebiotic administration, by modulating the gut microbiota, may represent a new therapeutic strategy for AD.

### FMT

Fecal microbiota transplantation is considered a safe therapeutic procedure with minor and transient side effects because of the introduction of live microorganisms and associated metabolites. It has been proven to be an effective treatment for recurrent *Clostridium difficile* infections (Khanna et al., 2017). In addition to its direct therapeutic effect in gastrointestinal diseases, FMT has also been shown to improve neurological and psychological symptoms by modulating the gut–brain axis. Hazan found that FMT can rapidly improve AD symptoms in a patient with AD with recurrent *Clostridium difficile* infection (Hazan, 2020). Using humanized mice by transplanting germ-free C57BL/6N mice with fecal samples from a patient with AD, Fujii et al. (2019) observed that these mice reproduce the characteristics of the donor microbiota, which affects mouse behavior. Zhan et al. (2018) found that cognitive dysfunction in SAMP8 mice can be attenuated by modulating the gut microbiota through FMT. In an APPswe/PS1dE9 transgenic (Tg) mouse model, Sun et al. (2019c) found that FMT treatment can improve cognitive deficits and reduce brain deposition of Aβ, which is accompanied by decreased phosphorylation of tau protein and the levels of Aβ40 and Aβ42, increased synaptic plasticity, and reversed changes in gut microbiota and SCFAs. Kim et al. (2020) also found that the transfer of a healthy microbiota alleviates Aβ, tau, and reactive glial pathology in the brain in an AD animal model. These studies indicate that FMT can correct gut microbiota dysbiosis and brain dysfunction in patients with AD rapidly and effectively, suggesting that restoration of gut microbial homeostasis by FMT may have beneficial effects on AD treatment. However, it is extremely important to properly screen the donor and fecal material to avoid contamination of the patient with pathogens that can lead to serious infections.

## Conclusion and Perspectives

There is no doubt that gut dysbiosis plays a vital role in modulating the microbiota–gut–brain axis and actively participates in the etiopathogenesis of AD. With the advent of multi-omics techniques, an increasing number of key functional bacteria associated with AD pathology and altered brain functions have been identified. These bacteria can be used as targets for the noninvasive diagnosis of AD and for further treatment. Gut microbiota interacts with AD pathogenesis via various pathways, such as Aβ abnormality, tau phosphorylation, neuroinflammation, neurotransmitter dysregulation, and oxidative stress. However, the exact roles and mechanisms of gut microbiota in patients with AD remain elusive. Most microbiome studies can only provide the associations between gut microbiota and AD, whereas the causal relationship between specific bacteria and AD pathology and brain dysfunctions cannot be illustrated. Thus, it is important to move microbiome studies from associations back to cultures to demonstrate causality. Clearly, culturomics have provided a novel strategy to obtain new bacterial taxa identified from microbiome studies. In addition, germ-free animals, specific gene knockout animals, or AD humanized animal models should be used to demonstrate the real roles and mechanisms of bacteria in AD. After clarifying the important roles of the gut bacteria in the pathophysiology of AD, novel intervention strategies targeting these bacteria can be developed for AD treatment, which can help prevent or delay AD onset or counteract its progression.

## Author Contributions

SW, XL, RJ, XY, and ZL discussed the contents, wrote, reviewed and edited the manuscript. The final manuscript was read and approved by all the authors.

## Conflict of Interest

The authors declare that the research was conducted in the absence of any commercial or financial relationships that could be construed as a potential conflict of interest.

## References

[B1] Agahi A. Hamidi G. A. Daneshvar R. Hamdieh M. Soheili M. Alinaghipour A. (2018). Does severity of Alzheimer’s disease contribute to its responsiveness to modifying gut microbiota? A double blind clinical trial. *Front. Neurol.* 9:662. 10.3389/fneur.2018.00662 30158897 PMC6104449

[B2] Akbari E. Asemi Z. Daneshvar Kakhaki R. Bahmani F. Kouchaki E. Tamtaji O. R. (2016). Effect of probiotic supplementation on cognitive function and metabolic status in Alzheimer’s disease: a randomized, double-blind and controlled trial. *Front. Aging Neurosci.* 8:256. 10.3389/fnagi.2016.00256 27891089 PMC5105117

[B3] Altaib H. Nakamura K. Abe M. Badr Y. Yanase E. Nomura I. (2021). Differences in the concentration of the fecal neurotransmitters GABA and glutamate are associated with microbial composition among healthy human subjects. *Microorganisms* 9:378. 10.3390/microorganisms9020378 33668550 PMC7918917

[B4] Alzheimer’s Association (2018). 2018 Alzheimer’s disease facts and figures. *Alzheimers Dement.* 14 367–425. 10.1016/j.jalz.2018.02.001

[B5] Andreu-Reinón M. E. Chirlaque M. D. Gavrila D. Amiano P. Mar J. Tainta M. (2021). Mediterranean diet and risk of dementia and Alzheimer’s disease in the EPIC-spain dementia cohort study. *Nutrients* 13:700. 10.3390/nu13020700 33671575 PMC7927039

[B6] Ashford M. T. Veitch D. P. Neuhaus J. Nosheny R. L. Tosun D. Weiner M. W. (2021). The search for a convenient procedure to detect one of the earliest signs of Alzheimer’s disease: a systematic review of the prediction of brain amyloid status. *Alzheimers Dement.* [Epub ahead of print]. 10.1002/alz.12253 33583100

[B7] Athari Nik Azm S. Djazayeri A. Safa M. Azami K. Ahmadvand B. Sabbaghziarani F. (2018). Lactobacilli and bifidobacteria ameliorate memory and learning deficits and oxidative stress in β-amyloid (1-42) injected rats. *Appl. Physiol. Nutr. Metab.* 43 718–726. 10.1139/apnm-2017-0648 29462572

[B8] Badshah H. Ali T. Rehman S. U. Amin F. -U. Ullah F. Kim T. H. (2016). Protective effect of lupeol against lipopolysaccharide-induced neuroinflammation via the p38/c-Jun N-terminal kinase pathway in the adult mouse brain. *J. Neuroimmune Pharm.* 11 48–60. 10.1007/s11481-015-9623-z 26139594

[B9] Barichello T. Generoso J. S. Simoes L. R. Faller C. J. Ceretta R. A. Petronilho F. (2015). Sodium butyrate prevents memory impairment by re-establishing BDNF and GDNF expression in experimental pneumococcal meningitis. *Mol. Neurobiol.* 52 734–740. 10.1007/s12035-014-8914-3 25284351

[B10] Beaumont M. Andriamihaja M. Lan A. Khodorova N. Audebert M. Blouin J.M. (2016). Detrimental effects for colonocytes of an increased exposure to luminal hydrogen sulfide: the adaptive response. *Free Radic. Biol. Med.* 93 155–164. 10.1016/j.freeradbiomed.2016.01.028 26849947

[B11] Berding K. Vlckova K. Marx W. Schellekens H. Stanton C. Clarke G. (2021). Diet and the microbiota-gut-brain axis: sowing the seeds of good mental health. *Adv. Nutr.* [Epub ahead of print]. 10.1093/advances/nmaa181 33693453 PMC8321864

[B12] Bonfili L. Cecarini V. Gogoi O. Gong C. Cuccioloni M. Angeletti M. (2020). Microbiota modulation as preventative and therapeutic approach in Alzheimer’s disease. *FEBS J.* 288 2836–2855. 10.1111/febs.15571 32969566

[B13] Brandscheid C. Schuck F. Reinhardt S. Schäfer K. H. Pietrzik C. U. Grimm M. (2017). Altered gut microbiome composition and tryptic activity of the 5xFAD Alzheimer’s mouse model. *J. Alzheimers Dis.* 56 775–788. 10.3233/jad-160926 28035935

[B14] Browne T. C. Mcquillan K. Mcmanus R. M. O’reilly J. A. Mills K. H. Lynch M. A. (2013). IFN-γ Production by amyloid β-specific Th1 cells promotes microglial activation and increases plaque burden in a mouse model of Alzheimer’s disease. *J. Immunol.* 190(5): 2241–2251. 10.4049/jimmunol.1200947 23365075

[B15] Burokas A. Moloney R. D. Dinan T. G. Cryan J. F. (2015). Microbiota regulation of the mammalian gut-brain axis. *Adv. Appl. Microbiol.* 91 1–62. 10.1016/bs.aambs.2015.02.001 25911232

[B16] Caini S. Bagnoli S. Palli D. Saieva C. Ceroti M. Bendinelli B. (2016). Total and cancer mortality in a cohort of ulcerative colitis and Crohn’s disease patients: the Florence inflammatory bowel disease study, 1978-2010. *Digest. Liver Dis.* 48 1162–1167. 10.1016/j.dld.2016.07.008 27481588

[B17] Calabrese F. Rossetti A. C. Racagni G. Gass P. Riva M. A. Molteni R. (2014). Brain-derived neurotrophic factor: a bridge between inflammation and neuroplasticity. *Front. Cell Neurosci.* 8:430. 10.3389/Fncel.2014.00430 25565964 PMC4273623

[B18] Cani P. D. (2017). Gut microbiota - at the intersection of everything? *Nat. Rev. Gastro Hepat.* 14 321–322. 10.1038/nrgastro.2017.54 28442782

[B19] Carranza-Naval M. J. Vargas-Soria M. Hierro-Bujalance C. Baena-Nieto G. Garcia-Alloza M. Infante-Garcia C. (2021). Alzheimer’s disease and diabetes: role of diet, microbiota and inflammation in preclinical models. *Biomolecules* 11:262. 10.3390/biom11020262 33578998 PMC7916805

[B20] Cattaneo A. Cattane N. Galluzzi S. Provasi S. Lopizzo N. Festari C. (2017). Association of brain amyloidosis with pro-inflammatory gut bacterial taxa and peripheral inflammation markers in cognitively impaired elderly. *Neurobiol. Aging* 49 60–68. 10.1016/j.neurobiolaging.2016.08.019 27776263

[B21] Cenit M. C. Nuevo I. C. Codoner-Franch P. Dinan T. G. Sanz Y. (2017). Gut microbiota and attention deficit hyperactivity disorder: new perspectives for a challenging condition. *Eur. Child Adoles. Psychol.* 26 1081–1092. 10.1007/s00787-017-0969-z 28289903

[B22] Chen C. Ahn E. H. Kang S. S. Liu X. Alam A. Ye K. (2020). Gut dysbiosis contributes to amyloid pathology, associated with C/EBPβ/AEP signaling activation in Alzheimer’s disease mouse model. *Sci. Adv.* 6:eaba0466. 10.1126/sciadv.aba0466 32832679 PMC7439296

[B23] Chen C. H. Lin C. L. Kao C. H. (2016). Irritable bowel syndrome is associated with an increased risk of Dementia: a nationwide population-based study. *PLoS One* 11:e0144589. 10.1371/journal.pone.0144589 26731277 PMC4701489

[B24] Chen Y. Fang L. Chen S. Zhou H. Fan Y. Lin L. (2020). Gut microbiome alterations precede cerebral amyloidosis and microglial pathology in a mouse model of Alzheimer’s disease. *Biomed Res. Int.* 2020:8456596. 10.1155/2020/8456596 32596386 PMC7273394

[B25] Cheng Y. Ling Z. Li L. (2020). The intestinal microbiota and colorectal cancer. *Front. Immunol.* 11:615056. 10.3389/fimmu.2020.615056 33329610 PMC7734048

[B26] Chin V. K. Yong V. C. Chong P. P. Amin Nordin S. Basir R. Abdullah M. (2020). Mycobiome in the gut: a multiperspective review. *Mediat. Inflamm.* 2020: 9560684. 10.1155/2020/9560684 32322167 PMC7160717

[B27] Clarke G. Sandhu K. V. Griffin B. T. Dinan T. G. Cryan J. F. Hyland N. P. (2019). Gut reactions: breaking down xenobiotic-microbiome interactions. *Pharmacol. Rev.* 71 198–224. 10.1124/pr.118.015768 30890566

[B28] Cryan J. F. Dinan T. G. (2012). Mind-altering microorganisms: the impact of the gut microbiota on brain and behaviour. *Nat. Rev. Neurosci.* 13 701–712. 10.1038/nrn3346 22968153

[B29] Cryan J. F. O’riordan K. J. Cowan C. S. M. Sandhu K. V. Bastiaanssen T. F. S. Boehme M. (2019). The microbiota-gut-brain axis. *Physiol. Rev.* 99 1877–2013. 10.1152/physrev.00018.2018 31460832

[B30] Cuervo-Zanatta D. Garcia-Mena J. Perez-Cruz C. (2021). Gut microbiota alterations and cognitive impairment are sexually dissociated in a transgenic mice model of Alzheimer’s disease. *J. Alzheimers Dis.* [Epub ahead of print]. 10.3233/jad-201367 33492296

[B31] Cui L. Hou N. N. Wu H. M. Zuo X. Lian Y. Z. Zhang C. N. (2020). Prevalence of Alzheimer’s disease and Parkinson’s disease in china: an updated systematical analysis. *Front. Aging Neurosci.* 12:603854. 10.3389/Fnagi.2020.603854 33424580 PMC7793643

[B32] Dalile B. Van Oudenhove L. Vervliet B. Verbeke K. (2019). The role of short-chain fatty acids in microbiota-gut-brain communication. *Nat. Rev. Gastro Hepat.* 16 461–478. 10.1038/s41575-019-0157-3 31123355

[B33] Dansokho C. Ait Ahmed D. Aid S. Toly-Ndour C. Chaigneau T. Calle V. (2016). Regulatory T cells delay disease progression in Alzheimer-like pathology. *Brain* 139(Pt 4), 1237–1251. 10.1093/brain/awv408 26912648

[B34] De-Paula J. R. V. Forlenza A. S. Forlenza O. V. (2018). Relevance of gutmicrobiota in cognition, behaviour and Alzheimer’s disease. *Pharmacol. Res.* 136 29–34. 10.1016/j.phrs.2018.07.007 30138667

[B35] Desbonnet L. Clarke G. Traplin A. O’sullivan O. Crispie F. Moloney R. D. (2015). Gut microbiota depletion from early adolescence in mice: implications for brain and behaviour. *Brain Behav. Immun.* 48 165–173. 10.1016/j.bbi.2015.04.004 25866195

[B36] Dewsbury L. S. Lim C. K. Steiner G. Z. (2021). The efficacy of ketogenic therapies in the clinical management of people with neurodegenerative disease: a systematic review. *Adv. Nutr.* [Epub ahead of print]. 10.1093/advances/nmaa180 33621313 PMC8321843

[B37] Diaz Heijtz R. Wang S. Anuar F. Qian Y. Björkholm B. Samuelsson A. (2011). Normal gut microbiota modulates brain development and behavior. *Proc. Natl. Acad. Sci. U.S.A.* 108 3047–3052. 10.1073/pnas.1010529108 21282636 PMC3041077

[B38] Dinan T. G. Cryan J. F. (2017). Gut feelings on parkinson’s and depression. *Cerebrum* 2017:cer-04–17. PMC5501039 28698775

[B39] Dodiya H. B. Frith M. Sidebottom A. Cao Y. Koval J. Chang E. (2020). Synergistic depletion of gut microbial consortia, but not individual antibiotics, reduces amyloidosis in APPPS1-21 Alzheimer’s transgenic mice. *Sci. Rep.* 10:8183. 10.1038/s41598-020-64797-5 32424118 PMC7235236

[B40] Dos Santos Guilherme M. Todorov H. Osterhof C. Möllerke A. Cub K. Hankeln T. (2020). Impact of acute and chronic Amyloid-β peptide exposure on gut microbial commensals in the mouse. *Front. Microbiol.* 11:1008. 10.3389/fmicb.2020.01008 32508799 PMC7251927

[B41] Dumitrescu L. Popescu-Olaru I. Cozma L. Tulba D. Hinescu M. E. Ceafalan L. C. (2018). Oxidative stress and the microbiota-gut-brain axis. *Oxid. Med Cell Longev.* 2018:2406594. 10.1155/2018/2406594 30622664 PMC6304899

[B42] Duranti S. Ruiz L. Lugli G. A. Tames H. Milani C. Mancabelli L. (2020). Bifidobacterium adolescentis as a key member of the human gut microbiota in the production of GABA. *Sci. Rep.* 10:14112. 10.1038/s41598-020-70986-z 32839473 PMC7445748

[B43] Erny D. De Angelis A. L. H. Jaitin D. Wieghofer P. Staszewski O. David E. (2015). Host microbiota constantly control maturation and function of microglia in the CNS. *Nat. Neurosci.* 18 965–977. 10.1038/nn.4030 26030851 PMC5528863

[B44] Eyerich K. Dimartino V. Cavani A. (2017). IL-17 and IL-22 in immunity: driving protection and pathology. *Eur. J. Immunol.* 47 607–614. 10.1002/eji.201646723 28295238

[B45] Feng Y. S. Tan Z. X. Wu L. Y. Dong F. Zhang F. (2020). The involvement of NLRP3 inflammasome in the treatment of Alzheimer’s disease. *Ageing Res. Rev.* 64:101192. 10.1016/j.arr.2020.101192 33059089

[B46] Fernando W. Martins I. J. Morici M. Bharadwaj P. Rainey-Smith S. R. Lim W. L. F. (2020). Sodium butyrate reduces brain Amyloid-β levels and improves cognitive memory performance in an Alzheimer’s disease transgenic mouse model at an early disease stage. *J. Alzheimers. Dis.* 74 91–99. 10.3233/jad-190120 31958090

[B47] Foster J. A. McVey Neufeld K. A. (2013). Gut-brain axis: how the microbiome influences anxiety and depression. *Trends Neurosci.* 36 305–312. 10.1016/j.tins.2013.01.005 23384445

[B48] Friedland R. P. Mcmillan J. D. Kurlawala Z. (2020). What are the molecular mechanisms by which functional bacterial amyloids influence amyloid beta deposition and neuroinflammation in neurodegenerative disorders? *Int. J. Mol. Sci.* 21 1652. 10.3390/ijms21051652 32121263 PMC7084682

[B49] Fujii Y. Nguyen T. T. T. Fujimura Y. Kameya N. Nakamura S. Arakawa K. (2019). Fecal metabolite of a gnotobiotic mouse transplanted with gut microbiota from a patient with Alzheimer’s disease. *Biosci. Biotechnol. Biochem.* 83 2144–2152. 10.1080/09168451.2019.1644149 31327302

[B50] Fulling C. Dinan T. G. Cryan J. F. (2019). Gut microbe to brain signaling: what happens in vagus. *Neuron* 101 998–1002. 10.1016/j.neuron.2019.02.008 30897366

[B51] Gerhardt S. Mohajeri M. H. (2018). Changes of colonic bacterial composition in Parkinson’s disease and other neurodegenerative diseases. *Nutrients* 10:708. 10.3390/nu10060708 29857583 PMC6024871

[B52] Ghosh T. S. Rampelli S. Jeffery I. B. Santoro A. Neto M. Capri M. (2020). Mediterranean diet intervention alters the gut microbiome in older people reducing frailty and improving health status: the NU-AGE 1-year dietary intervention across five European countries. *Gut* 69 1218–1228. 10.1136/gutjnl-2019-319654 32066625 PMC7306987

[B53] Gilbert J. A. Blaser M. J. Caporaso J. G. Jansson J. K. Lynch S. V. Knight R. (2018). Current understanding of the human microbiome. *Nat. Med.* 24 392–400. 10.1038/nm.4517 29634682 PMC7043356

[B54] Glenner G. G. Wong C. W. (1984). Alzheimer’s disease and Down’s syndrome: sharing of a unique cerebrovascular amyloid fibril protein. *Biochem. Biophys. Res. Commun.* 122 1131–1135. 10.1016/0006-291x(84)91209-9 6236805

[B55] Go J. Chang D. H. Ryu Y. K. Park H. Y. Lee I. B. Noh J. R. (2021). Human gut microbiota *Agathobaculum butyriciproducens* improves cognitive impairment in LPS-induced and APP/PS1 mouse models of Alzheimer’s disease. *Nutr. Res.* 86: 96–108. 10.1016/j.nutres.2020.12.010 33551257

[B56] Goedert M. (2018). Tau filaments in neurodegenerative diseases. *FEBS Lett.* 592 2383–2391. 10.1002/1873-3468.13108 29790176

[B57] Goswami C. Iwasaki Y. Yada T. (2018). Short-chain fatty acids suppress food intake by activating vagal afferent neurons. *J. Nutr. Biochem.* 57 130–135. 10.1016/j.jnutbio.2018.03.009 29702431

[B58] Govindpani K. Turner C. Waldvogel H. J. Faull R. L. M. Kwakowsky A. (2020). Impaired expression of GABA signaling components in the Alzheimer’s disease middle temporal gyrus. *Int. J. Mol. Sci.* 21:8704. 10.3390/ijms21228704 33218044 PMC7698927

[B59] Gribble F. M. Reimann F. (2019). Function and mechanisms of enteroendocrine cells and gut hormones in metabolism. *Nat. Rev. Endocrinol.* 15 226–237. 10.1038/s41574-019-0168-8 30760847

[B60] Griseri P. Patrone G. Puppo F. Romeo G. Ravazzolo R. Ceccherini I. (2003). Rescue of human RET gene expression by sodium butyrate: a novel powerful tool for molecular studies in Hirschsprung disease. *Gut* 52 1154–1158. 10.1136/gut.52.8.1154 12865274 PMC1773746

[B61] Guarner F. (2007). Hygiene, microbial diversity and immune regulation. *Curr. Opin. Gastroen.* 23 667–672. 10.1097/Mog.0b013e3282eeb43b 17906445

[B62] Guo M. Peng J. Huang X. Xiao L. Huang F. Zuo Z. (2021). Gut microbiome features of chinese patients newly diagnosed with Alzheimer’s disease or mild cognitive impairment. *J. Alzheimers Dis.* 80 299–310. 10.3233/jad-201040 33523001

[B63] Guo T. T. Zhang D. H. Zeng Y. Z. Huang T. Y. Xu H. X. Zhao Y. J. (2020). Molecular and cellular mechanisms underlying the pathogenesis of Alzheimer’s disease. *Mol. Neurodegener.* 15:40. 10.1186/S13024-020-00391-7 32677986 PMC7364557

[B64] Harach T. Marungruang N. Duthilleul N. Cheatham V. Mc Coy K. D. Frisoni G. (2017). Reduction of Abeta amyloid pathology in APPPS1 transgenic mice in the absence of gut microbiota. *Sci. Rep.* 7:41802. 10.1038/srep41802 28176819 PMC5297247

[B65] Haran J. P. Bhattarai S. K. Foley S. E. Dutta P. Ward D. V. Bucci V. (2019). Alzheimer’s disease microbiome is associated with dysregulation of the anti-inflammatory P-Glycoprotein pathway. *mBio* 10:e00632-19. 10.1128/mBio.00632-19 31064831 PMC6509190

[B66] Hardy J. Selkoe D. J. (2002). The amyloid hypothesis of Alzheimer’s disease: progress and problems on the road to therapeutics. *Science* 297 353–356. 10.1126/science.1072994 12130773

[B67] Hazan S. (2020). Rapid improvement in Alzheimer’s disease symptoms following fecal microbiota transplantation: a case report. *J. Int. Med. Res.* 48:300060520925930. 10.1177/0300060520925930 32600151 PMC7328362

[B68] He Y. Wu W. Zheng H. M. Li P. Mcdonald D. Sheng H. F. (2018). Regional variation limits applications of healthy gut microbiome reference ranges and disease models. *Nat. Med.* 24 1532–1535. 10.1038/s41591-018-0164-x 30150716

[B69] Heijtza R. D. Wang S. G. Anuar F. Qian Y. Bjorkholm B. Samuelsson A. (2011). Normal gut microbiota modulates brain development and behavior. *Proc. Natl. Acad. Sci. U.S.A.* 108 3047–3052. 10.1073/pnas.1010529108 21282636 PMC3041077

[B70] Heneka M. T. Kummer M. P. Stutz A. Delekate A. Schwartz S. Vieira-Saecker A. (2013). NLRP3 is activated in Alzheimer’s disease and contributes to pathology in APP/PS1 mice. *Nature* 493 674–678. 10.1038/nature11729 23254930 PMC3812809

[B71] Hill C. Guarner F. Reid G. Gibson G. R. Merenstein D. J. Pot B. (2014). Expert consensus document. The international scientific association for probiotics and prebiotics consensus statement on the scope and appropriate use of the term probiotic. *Nat. Rev. Gastroenterol. Hepatol.* 11 506–514. 10.1038/nrgastro.2014.66 24912386

[B72] Hill J. M. Lukiw W. J. (2015). Microbial-generated amyloids and Alzheimer’s disease (AD). *Front. Aging Neurosci.* 7:9. 10.3389/fnagi.2015.00009 25713531 PMC4322713

[B73] Ho L. Ono K. Tsuji M. Mazzola P. Singh R. Pasinetti G. M. (2018). Protective roles of intestinal microbiota derived short chain fatty acids in Alzheimer’s disease-type beta-amyloid neuropathological mechanisms. *Expert Rev. Neurother.* 18 83–90. 10.1080/14737175.2018.1400909 29095058 PMC5958896

[B74] Holtzman D.M. Morris J.C. Goate A.M. (2011). Alzheimer’s disease: the challenge of the second century. *Sci. Transl. Med.* 3:77sr71. 10.1126/scitranslmed.3002369 21471435 PMC3130546

[B75] Honarpisheh P. Reynolds C. R. Conesa M. P. B. Manchon J. F. M. Putluri N. Bhattacharjee M. B. (2020). Dysregulated gut homeostasis observed prior to the accumulation of the brain amyloid-beta in Tg2576 mice. *Int. J. Mol. Sci.* 21:1711. 10.3390/Ijms21051711 32138161 PMC7084806

[B76] Hrncir T. Hrncirova L. Kverka M. Tlaskalova-Hogenova H. (2019). The role of gut microbiota in intestinal and liver diseases. *Lab. Anim. Uk* 53 271–280. 10.1177/0023677218818605 30580671

[B77] Hu E. A. Wu A. Dearborn J. L. Gottesman R. F. Sharrett A. R. Steffen L. M. (2020). Adherence to dietary patterns and risk of incident dementia: findings from the atherosclerosis risk in communities study. *J. Alzheimers Dis.* 78 827–835. 10.3233/jad-200392 33044177 PMC7934551

[B78] Huai W. W. Zhao R. Song H. Zhao J. Zhang L. Zhang L. N. (2014). Aryl hydrocarbon receptor negatively regulates NLRP3 inflammasome activity by inhibiting NLRP3 transcription. *Nat. Commun.* 5:4738. 10.1038/Ncomms5738 25141024

[B79] Huseyin C. E. O’toole P. W. Cotter P. D. Scanlan P. D. (2017). Forgotten fungi-the gut mycobiome in human health and disease. *FEMS Microbiol. Rev.* 41 479–511. 10.1093/femsre/fuw047 28430946

[B80] Ivanov, Ii, Atarashi K. Manel N. Brodie E. L. Shima T. Karaoz U. (2009). Induction of intestinal Th17 cells by segmented filamentous bacteria. *Cell* 139 485–498. 10.1016/j.cell.2009.09.033 19836068 PMC2796826

[B81] Janeiro M. H. Ramírez M. J. Solas M. (2021). Dysbiosis and Alzheimer’s disease: cause or treatment opportunity? *Cell. Mol. Neurobiol.* [Epub ahead of print]. 10.1007/s10571-020-01024-9 33400081 PMC11441293

[B82] Javed I. Zhang Z. Adamcik J. Andrikopoulos N. Li Y. Otzen D. E. (2020). Accelerated amyloid beta pathogenesis by bacterial amyloid FapC. *Adv. Sci.* 7:2001299. 10.1002/advs.202001299 32999841 PMC7509637

[B83] Jia J. P. Wei C. B. Chen S. Q. Li F. Y. Tang Y. Qin W. (2018). The cost of Alzheimer’s disease in China and re-estimation of costs worldwide. *Alzheimers Dement.* 14 483–491. 10.1016/j.jalz.2017.12.006 29433981

[B84] Jiang C. M. Huang P. R. Liu Z. Zhao B. (2017). The gut microbiota and Alzheimer’s disease. *J. Alzheimers Dis.* 58 1–15. 10.3233/Jad-161141 28372330

[B85] Jiang H. Y. Ling Z. X. Zhang Y. H. Mao H. J. Ma Z. P. Yin Y. (2015). Altered fecal microbiota composition in patients with major depressive disorder. *Brain Behav. Immun.* 48 186–194. 10.1016/j.bbi.2015.03.016 25882912

[B86] Johnson K. A. Schultz A. Betensky R. A. Becker J. A. Sepulcre J. Rentz D. (2016). Tau positron emission tomographic imaging in aging and early Alzheimer disease. *Ann. Neurol.* 79 110–119. 10.1002/ana.24546 26505746 PMC4738026

[B87] Kennedy P. J. Cryan J. F. Dinan T. G. Clarke G. (2017). Kynurenine pathway metabolism and the microbiota-gut-brain axis. *Neuropharmacol* 112(Pt B), 399–412. 10.1016/j.neuropharm.2016.07.002 27392632

[B88] Khan M. S. H. Hegde V. (2020). Obesity and diabetes mediated chronic inflammation: a potential biomarker in Alzheimer’s disease. *J. Pers. Med.* 10:42. 10.3390/jpm10020042 32455946 PMC7354630

[B89] Khanna S. Vazquez-Baeza Y. González A. Weiss S. Schmidt B. Muñiz-Pedrogo D. A. (2017). Changes in microbial ecology after fecal microbiota transplantation for recurrent C. difficile infection affected by underlying inflammatory bowel disease. *Microbiome* 5:55. 10.1186/s40168-017-0269-3 28506317 PMC5433077

[B90] Kim C. S. Cha L. Sim M. Jung S. Chun W. Y. Baik H. W. (2021). Probiotic supplementation improves cognitive function and mood with changes in gut microbiota in community-dwelling older adults: a randomized, double-blind, placebo-controlled, multicenter trial. *J. Gerontol. A Biol. Sci. Med. Sci.* 76 32–40. 10.1093/gerona/glaa090 32300799 PMC7861012

[B91] Kim M. S. Kim Y. Choi H. Kim W. Park S. Lee D. (2020). Transfer of a healthy microbiota reduces amyloid and tau pathology in an Alzheimer’s disease animal model. *Gut* 69 283–294. 10.1136/gutjnl-2018-317431 31471351

[B92] Kobayashi Y. Kuhara T. Oki M. Xiao J. Z. (2019). Effects of Bifidobacterium breve A1 on the cognitive function of older adults with memory complaints: a randomised, double-blind, placebo-controlled trial. *Benef. Microbes* 10 511–520. 10.3920/bm2018.0170 31090457

[B93] Kobayashi Y. Sugahara H. Shimada K. Mitsuyama E. Kuhara T. Yasuoka A. (2017). Therapeutic potential of *Bifidobacterium breve* strain A1 for preventing cognitive impairment in Alzheimer’s disease. *Sci. Rep.* 7:13510. 10.1038/s41598-017-13368-2 29044140 PMC5647431

[B94] Konijnenberg E. Tomassen J. Den Braber A. Ten Kate M. Yaqub M. M. Mulder S. D. (2021). The onset of preclinical Alzheimer’s disease in monozygotic twins. *Ann. Neurol.* 89 987–1000. 10.1002/ana.26048 33583080 PMC8251701

[B95] Krüger J. F. Hillesheim E. Pereira A. Camargo C. Q. Rabito E. I. (2021). Probiotics for dementia: a systematic review and meta-analysis of randomized controlled trials. *Nutr. Rev.* 79 160–170. 10.1093/nutrit/nuaa037 32556236

[B96] Kumar D.K. Choi S.H. Washicosky K.J. Eimer W.A. Tucker S. Ghofrani J. (2016). Amyloid-β peptide protects against microbial infection in mouse and worm models of Alzheimer’s disease. *Sci. Transl. Med.* 8:340ra372. 10.1126/scitranslmed.aaf1059 27225182 PMC5505565

[B97] Larbi A. Pawelec G. Witkowski J. M. Schipper H. M. Derhovanessian E. Goldeck D. (2009). Dramatic shifts in circulating CD4 but not CD8 T cell subsets in mild Alzheimer’s disease. *J. Alzheimers Dis.* 17 91–103. 10.3233/jad-2009-1015 19494434

[B98] Larsen N. Vogensen F. K. Van Den Berg F. W. Nielsen D. S. Andreasen A. S. Pedersen B. K. (2010). Gut microbiota in human adults with type 2 diabetes differs from non-diabetic adults. *PLoS One* 5:e9085. 10.1371/journal.pone.0009085 20140211 PMC2816710

[B99] Lee H. J. Lee K. E. Kim J. K. Kim D. H. (2019). Suppression of gut dysbiosis by *Bifidobacterium longum* alleviates cognitive decline in 5XFAD transgenic and aged mice. *Sci. Rep.* 9:11814. 10.1038/s41598-019-48342-7 31413350 PMC6694197

[B100] Lee Y. S. Lai D. M. Huang H. J. Lee-Chen G. J. Chang C. H. Hsieh-Li H. M. (2021). Prebiotic lactulose ameliorates the cognitive deficit in Alzheimer’s disease mouse model through macroautophagy and chaperone-mediated autophagy pathways. *J. Agric. Food Chem.* 69 2422–2437. 10.1021/acs.jafc.0c07327 33617267

[B101] Leuzy A. Ashton N. J. Mattsson-Carlgren N. Dodich A. Boccardi M. Corre J. (2021). 2020 update on the clinical validity of cerebrospinal fluid amyloid, tau, and phospho-tau as biomarkers for Alzheimer’s disease in the context of a structured 5-phase development framework. *Eur. J. Nucl. Med. Mol. Imaging* [Epub ahead of print]. 10.1007/s00259-021-05258-7 33674895 PMC8175301

[B102] Li B. He Y. Ma J. Huang P. Du J. Cao L. (2019). Mild cognitive impairment has similar alterations as Alzheimer’s disease in gut microbiota. *Alzheimers Dement.* 15 1357–1366. 10.1016/j.jalz.2019.07.002 31434623

[B103] Li H. Sun J. Wang F. Ding G. Chen W. Fang R. (2016). Sodium butyrate exerts neuroprotective effects by restoring the blood-brain barrier in traumatic brain injury mice. *Brain Res.* 1642 70–78. 10.1016/j.brainres.2016.03.031 27017959

[B104] Li J. M. Yu R. Zhang L. P. Wen S. Y. Wang S. J. Zhang X. Y. (2019). Dietary fructose-induced gut dysbiosis promotes mouse hippocampal neuroinflammation: a benefit of short-chain fatty acids. *Microbiome* 7:98. 10.1186/s40168-019-0713-7 31255176 PMC6599330

[B105] Li X. V. Leonardi I. Iliev I. D. (2019). Gut mycobiota in immunity and inflammatory disease. *Immunity* 50 1365–1379. 10.1016/j.immuni.2019.05.023 31216461 PMC6585451

[B106] Lin T. L. Shu C. C. Chen Y. M. Lu J. J. Wu T. S. Lai W. F. (2020). Like cures like: pharmacological activity of anti-inflammatory lipopolysaccharides from gut microbiome. *Front. Pharmacol.* 11:554. 10.3389/fphar.2020.00554 32425790 PMC7212368

[B107] Ling Z. Liu X. Jia X. Cheng Y. Luo Y. Yuan L. (2014). Impacts of infection with different toxigenic *Clostridium difficile* strains on faecal microbiota in children. *Sci. Rep.* 4:7485. 10.1038/srep07485 25501371 PMC4265774

[B108] Ling Z. Shao L. Liu X. Cheng Y. Yan C. Mei Y. (2019). Regulatory T Cells and plasmacytoid dendritic cells within the tumor microenvironment in gastric cancer are correlated with gastric microbiota dysbiosis: a preliminary study. *Front. Immunol.* 10:533. 10.3389/fimmu.2019.00533 30936882 PMC6433099

[B109] Ling Z. Zhu M. Liu X. Shao L. Cheng Y. Yan X. (2021a). Fecal fungal dysbiosis in chinese patients with Alzheimer’s disease. *Front. Cell Dev. Biol.* 8:631460. 10.3389/fcell.2020.631460 33585471 PMC7876328

[B110] Ling Z. Zhu M. Yan X. Cheng Y. Shao L. Liu X. (2021b). Structural and functional dysbiosis of fecal microbiota in chinese patients with Alzheimer’s disease. *Front. Cell Dev. Biol.* 8:634069. 10.3389/fcell.2020.634069 33614635 PMC7889981

[B111] Liu J. Sun J. Wang F. Yu X. Ling Z. Li H. (2015). Neuroprotective effects of clostridium butyricum against vascular dementia in mice via metabolic butyrate. *Biomed Res. Int.* 2015:412946. 10.1155/2015/412946 26523278 PMC4615854

[B112] Liu M. Hu R. Guo Y. Sun W. Li J. Fan M. (2020). Influence of Lactobacillus reuteri SL001 on intestinal microbiota in AD model mice and C57BL/6 mice. *Sheng Wu Gong Cheng Xue Bao* 36 1887–1898. 10.13345/j.cjb.200024 33164464

[B113] Liu P. Wu L. Peng G. Han Y. Tang R. Ge J. (2019). Altered microbiomes distinguish Alzheimer’s disease from amnestic mild cognitive impairment and health in a Chinese cohort. *Brain Behav. Immun.* 80 633–643. 10.1016/j.bbi.2019.05.008 31063846

[B114] Liu X. Cheng Y. Shao L. Ling Z. (2020). Alterations of the predominant fecal microbiota and disruption of the gut mucosal barrier in patients with early-stage colorectal cancer. *Biomed Res. Int.* 2020:2948282. 10.1155/2020/2948282 32280686 PMC7114766

[B115] Liu X. Shao L. Liu X. Ji F. Mei Y. Cheng Y. (2019). Alterations of gastric mucosal microbiota across different stomach microhabitats in a cohort of 276 patients with gastric cancer. *EBioMedicine* 40 336–348. 10.1016/j.ebiom.2018.12.034 30584008 PMC6412016

[B116] Liu Z. Li L. Ma S. Ye J. Zhang H. Li Y. (2020). High-dietary fiber intake alleviates antenatal obesity-induced postpartum depression: roles of gut microbiota and microbial metabolite short-chain fatty acid involved. *J. Agric. Food Chem.* 68 13697–13710. 10.1021/acs.jafc.0c04290 33151669

[B117] Lowe P. P. Gyongyosi B. Satishchandran A. Iracheta-Vellve A. Cho Y. Ambade A. (2018). Reduced gut microbiome protects from alcohol-induced neuroinflammation and alters intestinal and brain inflammasome expression. *J. Neuroinflamm.* 15:298. 10.1186/S12974-018-1328-9 30368255 PMC6203993

[B118] Ma D. Wang A. C. Parikh I. Green S. J. Hoffman J. D. Chlipala G. (2018). Ketogenic diet enhances neurovascular function with altered gut microbiome in young healthy mice. *Sci. Rep.* 8:6670. 10.1038/s41598-018-25190-5 29703936 PMC5923270

[B119] Madore C. Yin Z. R. Leibowitz J. Butovsky O. (2020). Microglia, lifestyle stress, and neurodegeneration. *Immunity* 52 222–240. 10.1016/j.immuni.2019.12.003 31924476 PMC7234821

[B120] Manyevitch R. Protas M. Scarpiello S. Deliso M. Bass B. Nanajian A. (2018). Evaluation of metabolic and synaptic dysfunction hypotheses of Alzheimer’s disease (AD): a meta-analysis of CSF markers. *Curr. Alzheimer Res.* 15 164–181. 10.2174/1567205014666170921122458 28933272 PMC5769087

[B121] Marizzoni M. Cattaneo A. Mirabelli P. Festari C. Lopizzo N. Nicolosi V. (2020). Short-chain fatty acids and lipopolysaccharide as mediators between gut dysbiosis and amyloid pathology in Alzheimer’s disease. *J. Alzheimers Dis.* 78 683–697. 10.3233/jad-200306 33074224

[B122] Markowiak-Kopeć P. Śliżewska K. (2020). The effect of probiotics on the production of short-chain fatty acids by human intestinal microbiome. *Nutrients* 12:1107. 10.3390/nu12041107 32316181 PMC7230973

[B123] Matheoud D. Cannon T. Voisin A. Penttinen A. -M. Ramet L. Fahmy A. M. (2019). Intestinal infection triggers Parkinson’s disease-like symptoms in Pink1–/– mice. *Nature* 571 565–569. 10.1038/s41586-019-1405-y 31316206

[B124] Mattsson-Carlgren N. Andersson E. Janelidze S. Ossenkoppele R. Insel P. Strandberg O. (2020). Aβ deposition is associated with increases in soluble and phosphorylated tau that precede a positive Tau PET in Alzheimer’s disease. *Sci. Adv.* 6:eaaz2387. 10.1126/sciadv.aaz2387 32426454 PMC7159908

[B125] Mccarville J. L. Chen G. Y. Cuevas V. D. Troha K. Ayres J. S. (2020). Microbiota metabolites in health and disease. *Annu. Rev. Immunol.* 38 147–170. 10.1146/annurev-immunol-071219-125715 32340573

[B126] Montoya A. Elgueta D. Campos J. Chovar O. Falcón P. Matus S. (2019). Dopamine receptor D3 signalling in astrocytes promotes neuroinflammation. *J. Neuroinflamm.* 16:258. 10.1186/s12974-019-1652-8 31810491 PMC6896356

[B127] Morais L. H. Schreiber H. L. T. Mazmanian S. K. (2021). The gut microbiota-brain axis in behaviour and brain disorders. *Nat. Rev. Microbiol.* 19 241–255. 10.1038/s41579-020-00460-0 33093662

[B128] Morris G. Berk M. Carvalho A. Caso J. Sanz Y. Walder K. (2017). The role of the microbial metabolites including tryptophan catabolites and short chain fatty acids in the pathophysiology of immune-inflammatory and neuroimmune disease. *Mol. Neurobiol.* 54 4432–4451. 10.1007/s12035-016-0004-2 27349436

[B129] Morrison D. J. Preston T. (2016). Formation of short chain fatty acids by the gut microbiota and their impact on human metabolism. *Gut Microbes* 7 189–200. 10.1080/19490976.2015.1134082 26963409 PMC4939913

[B130] Nagpal R. Neth B. J. Wang S. Craft S. Yadav H. (2019). Modified Mediterranean-ketogenic diet modulates gut microbiome and short-chain fatty acids in association with Alzheimer’s disease markers in subjects with mild cognitive impairment. *EBioMedicine* 47 529–542. 10.1016/j.ebiom.2019.08.032 31477562 PMC6796564

[B131] Ni J. Wu G. D. Albenberg L. Tomov V. T. (2017). Gut microbiota and IBD: causation or correlation? *Nat. Rev. Gastroenterol. Hepatol.* 14 573–584. 10.1038/nrgastro.2017.88 28743984 PMC5880536

[B132] Nishiwaki H. Ito M. Ishida T. Hamaguchi T. Maeda T. Kashihara K. (2020). Meta-analysis of gut dysbiosis in Parkinson’s disease. *Mov. Disord.* 35 1626–1635. 10.1002/mds.28119 32557853

[B133] Oh J. Eser R. A. Ehrenberg A. J. Morales D. Petersen C. Kudlacek J. (2019). Profound degeneration of wake-promoting neurons in Alzheimer’s disease. *Alzheimers Dement.* 15 1253–1263. 10.1016/j.jalz.2019.06.3916 31416793 PMC6801040

[B134] Ohta S. (2014). Molecular hydrogen as a preventive and therapeutic medical gas: initiation, development and potential of hydrogen medicine. *Pharmacol. Ther.* 144 1–11. 10.1016/j.pharmthera.2014.04.006 24769081

[B135] Oleskin A. V. Shenderov B. A. (2016). Neuromodulatory effects and targets of the SCFAs and gasotransmitters produced by the human symbiotic microbiota. *Microb. Ecol. Health Dis.* 27:30971. 10.3402/mehd.v27.30971 27389418 PMC4937721

[B136] Oleskin A. V. Shenderov B. A. (2019). Probiotics and psychobiotics: the role of microbial neurochemicals. *Probiot. Antimicrob. Proteins* 11 1071–1085. 10.1007/s12602-019-09583-0 31493127

[B137] Oli M. W. Otoo H. N. Crowley P. J. Heim K. P. Nascimento M. M. Ramsook C. B. (2012). Functional amyloid formation by Streptococcus mutans. *Microbiology* 158(Pt 12), 2903–2916. 10.1099/mic.0.060855-0 23082034 PMC4083658

[B138] Paranjapye N. Daggett V. (2018). De novo designed α-sheet peptides inhibit functional amyloid formation of streptococcus mutans biofilms. *J. Mol. Biol.* 430 3764–3773. 10.1016/j.jmb.2018.07.005 30006266 PMC6168415

[B139] Park A.-M. Omura S. Fujita M. Sato F. Tsunoda I. (2017). *Helicobacter pyloriand* gut microbiota in multiple sclerosis versus Alzheimer’s disease: 10 pitfalls of microbiome studies. *Clin. Exp. Neuroimmunol.* 8 215–232. 10.1111/cen3.12401 29158778 PMC5693366

[B140] Park J. Y. Choi J. Lee Y. Lee J. E. Lee E. H. Kwon H. J. (2017). Metagenome analysis of bodily microbiota in a mouse model of alzheimer disease using bacteria-derived membrane vesicles in blood. *Exp. Neurobiol.* 26 369–379. 10.5607/en.2017.26.6.369 29302204 PMC5746502

[B141] Park S. Zhang T. Wu X. Yi Qiu J. (2020). Ketone production by ketogenic diet and by intermittent fasting has different effects on the gut microbiota and disease progression in an Alzheimer’s disease rat model. *J. Clin. Biochem. Nutr.* 67 188–198. 10.3164/jcbn.19-87 33041517 PMC7533860

[B142] Pellegrini C. Antonioli L. Calderone V. Colucci R. Fornai M. Blandizzi C. (2020). Microbiota-gut-brain axis in health and disease: is NLRP3 inflammasome at the crossroads of microbiota-gut-brain communications? *Prog. Neurobiol.* 191:101806. 10.1016/j.pneurobio.2020.101806 32473843

[B143] Pellegrini C. Antonioli L. Lopez-Castejon G. Blandizzi C. Fornai M. (2017). Canonical and non-canonical activation of NLRP3 inflammasome at the crossroad between immune tolerance and intestinal inflammation. *Front. Immunol.* 8:36. 10.3389/Fimmu.2017.00036 28179906 PMC5263152

[B144] Pellicanò M. Larbi A. Goldeck D. Colonna-Romano G. Buffa S. Bulati M. (2012). Immune profiling of Alzheimer patients. *J. Neuroimmunol.* 242 52–59. 10.1016/j.jneuroim.2011.11.005 22153977

[B145] Piancone F. La Rosa F. Marventano I. Saresella M. Clerici M. (2021). The role of the inflammasome in neurodegenerative diseases. *Molecules* 26:953. 10.3390/molecules26040953 33670164 PMC7916884

[B146] Pistollato F. Cano S. S. Elio I. Vergara M. M. Giampieri F. Battino M. (2016). Role of gut microbiota and nutrients in amyloid formation and pathogenesis of Alzheimer disease. *Nutr. Rev.* 74 624–634. 10.1093/nutrit/nuw023 27634977

[B147] Pokusaeva K. Johnson C. Luk B. Uribe G. Fu Y. Oezguen N. (2017). GABA-producing *Bifidobacterium dentium* modulates visceral sensitivity in the intestine. *Neurogastroent. Motil.* 29:e12904. 10.1111/nmo.12904 27458085 PMC5195897

[B148] Qian X. H. Song X. X. Liu X. L. Chen S. D. Tang H. D. (2021). Inflammatory pathways in Alzheimer’s disease mediated by gut microbiota. *Ageing Res. Rev.* 68:101317. 10.1016/j.arr.2021.101317 33711509

[B149] Qureshi I. A. Mehler M. F. (2013). Towards a ‘systems’-level understanding of the nervous system and its disorders. *Trends Neurosci.* 36 674–684. 10.1016/j.tins.2013.07.003 23988221 PMC3818389

[B150] Ritchie C. Smailagic N. Noel-Storr A. H. Ukoumunne O. Ladds E. C. Martin S. (2017). CSF tau and the CSF tau/ABeta ratio for the diagnosis of Alzheimer’s disease dementia and other dementias in people with mild cognitive impairment (MCI). *Cochrane Database Syst. Rev.* 3:CD010803. 10.1002/14651858.CD010803.pub2 28328043 PMC6464349

[B151] Rogers G. B. Keating D. J. Young R. L. Wong M. L. Licinio J. Wesselingh S. (2016). From gut dysbiosis to altered brain function and mental illness: mechanisms and pathways. *Mol. Psychiatr.* 21 738–748. 10.1038/mp.2016.50 27090305 PMC4879184

[B152] Rutsch A. Kantsjö J. B. Ronchi F. (2020). The gut-brain axis: how microbiota and host inflammasome influence brain physiology and pathology. *Front. Immunol.* 11:604179. 10.3389/fimmu.2020.604179 33362788 PMC7758428

[B153] Sam Q. H. Chang M. W. Chai L. Y. (2017). The fungal mycobiome and its interaction with gut bacteria in the host. *Int. J. Mol. Sci.* 18:330. 10.3390/ijms18020330 28165395 PMC5343866

[B154] Samuel B. S. Shaito A. Motoike T. Rey F. E. Backhed F. Manchester J. K. (2008). Effects of the gut microbiota on host adiposity are modulated by the short-chain fatty-acid binding G protein-coupled receptor, Gpr41. *Proc. Natl. Acad. Sci. U.S.A.* 105 16767–16772. 10.1073/pnas.0808567105 18931303 PMC2569967

[B155] Saresella M. Calabrese E. Marventano I. Piancone F. Gatti A. Calvo M. G. (2010). PD1 negative and PD1 positive CD4+ T regulatory cells in mild cognitive impairment and Alzheimer’s disease. *J. Alzheimers Dis.* 21 927–938. 10.3233/jad-2010-091696 20634592

[B156] Sarkar A. Lehto S. M. Harty S. Dinan T. G. Cryan J. F. Burnet P. W. J. (2016). Psychobiotics and the manipulation of bacteria-gut-brain signals. *Trends Neurosci.* 39 763–781. 10.1016/j.tins.2016.09.002 27793434 PMC5102282

[B157] Scheltens P. De Strooper B. Kivipelto M. Holstege H. Chételat G. Teunissen C. E. (2021). Alzheimer’s disease. *Lancet* 397 1577–1590 10.1016/s0140-6736(20)32205-4 33667416 PMC8354300

[B158] Sender R. Fuchs S. Milo R. (2016). Revised estimates for the number of human and bacteria cells in the body. *PLoS Biol.* 14:e1002533. 10.1371/journal.pbio.1002533 27541692 PMC4991899

[B159] Seo D. O. Holtzman D. M. (2020). Gut microbiota: from the forgotten organ to a potential key player in the pathology of Alzheimer’s disease. *J. Gerontol. A Biol. Sci. Med. Sci.* 75 1232–1241. 10.1093/gerona/glz262 31738402 PMC7302187

[B160] Shabbir U. Arshad M. S. Sameen A. Oh D. H. (2021). Crosstalk between gut and brain in Alzheimer’s disease: the role of gut microbiota modulation strategies. *Nutrients* 13:690. 10.3390/nu13020690 33669988 PMC7924846

[B161] Shamsipour S. Sharifi G. Taghian F. (2021). An 8-week administration of *Bifidobacterium bifidum* and *Lactobacillus plantarum* combined with exercise training alleviates neurotoxicity of Aβ and spatial learning via acetylcholine in alzheimer rat model. *J. Mol. Neurosci.* [Epub ahead of print]. 10.1007/s12031-021-01812-y 33715084

[B162] Shen H. Guan Q. Zhang X. Yuan C. Tan Z. Zhai L. (2020). New mechanism of neuroinflammation in Alzheimer’s disease: the activation of NLRP3 inflammasome mediated by gut microbiota. *Prog. Neuropsychopharmacol. Biol. Psychiatry* 100:109884. 10.1016/j.pnpbp.2020.109884 32032696

[B163] Silver R. Curley J. P. (2013). Mast cells on the mind: new insights and opportunities. *Trends Neurosci.* 36 513–521. 10.1016/j.tins.2013.06.001 23845731

[B164] Sochocka M. Donskow-Łysoniewska K. Diniz B. S. Kurpas D. Brzozowska E. Leszek J. (2019). The gut microbiome alterations and inflammation-driven pathogenesis of Alzheimer’s disease-a critical review. *Mol. Neurobiol.* 56 1841–1851. 10.1007/s12035-018-1188-4 29936690 PMC6394610

[B165] Sokol H. Pigneur B. Watterlot L. Lakhdari O. Bermudez-Humaran L. G. Gratadoux J. J. (2008). *Faecalibacterium prausnitzii* is an anti-inflammatory commensal bacterium identified by gut microbiota analysis of Crohn disease patients. *Proc. Natl. Acad. Sci. U.S.A.* 105 16731–16736. 10.1073/pnas.0804812105 18936492 PMC2575488

[B166] Song J. H. Lee J. W. Shim B. Lee C. Y. Choi S. Kang C. (2013). Glycyrrhizin alleviates neuroinflammation and memory deficit induced by systemic lipopolysaccharide treatment in mice. *Molecules* 18 15788–15803. 10.3390/molecules181215788 24352029 PMC6269849

[B167] Song L. M. Pei L. Yao S. L. Wu Y. Shang Y. (2017). NLRP3 inflammasome in neurological diseases, from functions to therapies. *Front. Cell Neurosci.* 11:63. 10.3389/Fncel.2017.00063 28337127 PMC5343070

[B168] Spitzer P. Condic M. Herrmann M. Oberstein T. J. Scharin-Mehlmann M. Gilbert D. F. (2016). Amyloidogenic amyloid-β-peptide variants induce microbial agglutination and exert antimicrobial activity. *Sci. Rep.* 6:32228. 10.1038/srep32228 27624303 PMC5021948

[B169] Stanciu G. D. Luca A. Rusu R. N. Bild V. Beschea Chiriac S. I. Solcan C. (2019). Alzheimer’s disease pharmacotherapy in relation to cholinergic system involvement. *Biomolecules* 10:40. 10.3390/biom10010040 31888102 PMC7022522

[B170] Sun B. L. Li W. W. Wang J. Xu Y. L. Sun H. L. Tian D. Y. (2019a). Gut microbiota alteration and its time course in a tauopathy mouse model. *J. Alzheimers Dis.* 70 399–412. 10.3233/jad-181220 31177213

[B171] Sun J. Li H. Jin Y. Yu J. Mao S. Su K. P. (2021). Probiotic *Clostridium butyricum* ameliorated motor deficits in a mouse model of Parkinson’s disease via gut microbiota-GLP-1 pathway. *Brain Behav. Immun.* 91 703–715. 10.1016/j.bbi.2020.10.014 33148438

[B172] Sun J. Liu S. Ling Z. Wang F. Ling Y. Gong T. (2019b). Fructooligosaccharides ameliorating cognitive deficits and neurodegeneration in APP/PS1 transgenic mice through modulating gut microbiota. *J. Agric. Food Chem.* 67 3006–3017. 10.1021/acs.jafc.8b07313 30816709

[B173] Sun J. Wang F. Hong G. Pang M. Xu H. Li H. (2016a). Antidepressant-like effects of sodium butyrate and its possible mechanisms of action in mice exposed to chronic unpredictable mild stress. *Neurosci. Lett.* 618 159–166. 10.1016/j.neulet.2016.03.003 26957230

[B174] Sun J. Wang F. Li H. Zhang H. Jin J. Chen W. (2015). Neuroprotective effect of sodium butyrate against cerebral ischemia/reperfusion injury in mice. *Biomed Res. Int.* 2015:395895. 10.1155/2015/395895 26064905 PMC4439479

[B175] Sun J. Wang F. Ling Z. Yu X. Chen W. Li H. (2016b). Clostridium butyricum attenuates cerebral ischemia/reperfusion injury in diabetic mice via modulation of gut microbiota. *Brain Res.* 1642 180–188. 10.1016/j.brainres.2016.03.042 27037183

[B176] Sun J. Xu J. Ling Y. Wang F. Gong T. Yang C. (2019c). Fecal microbiota transplantation alleviated Alzheimer’s disease-like pathogenesis in APP/PS1 transgenic mice. *Transl. Psychiatry* 9:189. 10.1038/s41398-019-0525-3 31383855 PMC6683152

[B177] Sun J. Xu J. Yang B. Chen K. Kong Y. Fang N. (2020a). Effect of *Clostridium butyricum* against microglia-mediated neuroinflammation in Alzheimer’s disease via regulating gut microbiota and metabolites butyrate. *Mol. Nutr. Food Res.* 64:e1900636. 10.1002/mnfr.201900636 31835282

[B178] Sun J. Yuan B. Wu Y. Gong Y. Guo W. Fu S. (2020b). Sodium butyrate protects N2a cells against Aβ toxicity in vitro. *Mediat. Inflamm.* 2020:7605160. 10.1155/2020/7605160 32377164 PMC7180402

[B179] Sun Y. Sommerville N. R. Liu J. Y. H. Ngan M. P. Poon D. Ponomarev E. D. (2020c). Intra-gastrointestinal amyloid-β1-42 oligomers perturb enteric function and induce Alzheimer’s disease pathology. *J. Physiol.* 598 4209–4223. 32617993 10.1113/JP279919 PMC7586845

[B180] Sun Y. Y. Sommerville N. R. Liu J. Y. H. Ngan M. P. Poon D. Ponomarev E. D. (2020e). Intra-gastrointestinal amyloid-beta 1-42 oligomers perturb enteric function and induce Alzheimer’s disease pathology. *J. Physiol. London* 598 4209–4223. 10.1113/JP279919 32617993 PMC7586845

[B181] Sun Y.X. Jiang X.J. Lu B. Gao Q. Chen Y.F. Wu D.B. (2020d). Roles of gut microbiota in pathogenesis of Alzheimer’s disease and therapeutic effects of Chinese medicine. *Chin. J. Integr. Med.* [Epub ahead of print]. 10.1007/s11655-020-3274-5 32876860

[B182] Szabo C. (2010). Gaseotransmitters: new frontiers for translational science. *Sci. Transl. Med.* 2:59ps54. 10.1126/scitranslmed.3000721 21106939 PMC3038605

[B183] Tamtaji O. R. Heidari-Soureshjani R. Mirhosseini N. Kouchaki E. Bahmani F. Aghadavod E. (2019). Probiotic and selenium co-supplementation, and the effects on clinical, metabolic and genetic status in Alzheimer’s disease: a randomized, double-blind, controlled trial. *Clin. Nutr.* 38 2569–2575. 10.1016/j.clnu.2018.11.034 30642737

[B184] Thelen M. Brown-Borg H. M. (2020). Does diet have a role in the treatment of Alzheimer’s disease? *Front. Aging Neurosci.* 12:617071. 10.3389/fnagi.2020.617071 33424583 PMC7785773

[B185] Valls-Pedret C. Sala-Vila A. Serra-Mir M. Corella D. De La Torre R. Martínez-González M. (2015). Mediterranean diet and age-related cognitive decline: a randomized clinical trial. *JAMA Intern. Med.* 175 1094–1103. 10.1001/jamainternmed.2015.1668 25961184

[B186] Van Tongeren S. P. Slaets J. P. J. Harmsen H. J. M. Welling G. W. (2005). Fecal microbiota composition and frailty. *Appl. Environ. Microbiol.* 71 6438–6442. 10.1128/Aem.71.10.6438-6442.2005 16204576 PMC1265947

[B187] Velazquez R. Ferreira E. Knowles S. Fux C. Rodin A. Winslow W. (2019). Lifelong choline supplementation ameliorates Alzheimer’s disease pathology and associated cognitive deficits by attenuating microglia activation. *Aging Cell* 18:e13037. 10.1111/acel.13037 31560162 PMC6826123

[B188] Venegas C. Kumar S. Franklin B. S. Dierkes T. Brinkschulte R. Tejera D. (2017). Microglia-derived ASC specks cross-seed amyloid-beta in Alzheimer’s disease. *Nature* 552 355–361. 10.1038/nature25158 29293211

[B189] Vogt N. M. Kerby R. L. Dill-Mcfarland K. A. Harding S. J. Merluzzi A. P. Johnson S. C. (2017). Gut microbiome alterations in Alzheimer’s disease. *Sci. Rep.* 7:13537. 10.1038/s41598-017-13601-y 29051531 PMC5648830

[B190] Vogt N. M. Romano K. A. Darst B. F. Engelman C. D. Johnson S. C. Carlsson C. M. (2018). The gut microbiota-derived metabolite trimethylamine N-oxide is elevated in Alzheimer’s disease. *Alzheimers Res. Ther.* 10:124. 10.1186/s13195-018-0451-2 30579367 PMC6303862

[B191] Vonderwalde I. Finlayson-Trick E. (2021). Gut amyloid-β induces cognitive deficits and Alzheimer’s disease-related histopathology in a mouse model. *J. Physiol.* 599 15–16. 10.1113/jp280624 32985682

[B192] Wang F. Xu T. Zhang Y. Zheng T. He Y. He F. (2020). Long-term combined administration of *Bifidobacterium bifidum* TMC3115 and *Lactobacillus plantarum* 45 alleviates spatial memory impairment and gut dysbiosis in APP/PS1 mice. *FEMS Microbiol. Lett.* 367:fnaa048. 10.1093/femsle/fnaa048 32239209

[B193] Wang Q. J. Shen Y. E. Wang X. Fu S. Zhang X. Zhang Y. N. (2020). Concomitant memantine and Lactobacillus plantarum treatment attenuates cognitive impairments in APP/PS1 mice. *Aging* 12 628–649. 10.18632/aging.102645 31907339 PMC6977692

[B194] Wang S. H. Li J. Xia W. Geng M. Y. (2007). A marine-derived acidic oligosaccharide sugar chain specifically inhibits neuronal cell injury mediated by beta-amyloid-induced astrocyte activation in vitro. *Neurol. Res.* 29 96–102. 10.1179/174313206x152483 17427283

[B195] Wang W. Chen L. P. Zhou R. Wang X. B. Song L. Huang S. (2014). Increased proportions of bifidobacterium and the lactobacillus group and loss of butyrate-producing bacteria in inflammatory bowel disease. *J. Clin. Microbiol.* 52 398–406. 10.1128/Jcm.01500-13 24478468 PMC3911339

[B196] Wang X. Sun G. Feng T. Zhang J. Huang X. Wang T. (2019). Sodium oligomannate therapeutically remodels gut microbiota and suppresses gut bacterial amino acids-shaped neuroinflammation to inhibit Alzheimer’s disease progression. *Cell Res.* 29 787–803. 10.1038/s41422-019-0216-x 31488882 PMC6796854

[B197] Wang X. L. Zeng J. Yang Y. Xiong Y. Zhang Z. H. Qiu M. (2015). *Helicobacter pylori* filtrate induces Alzheimer-like tau hyperphosphorylation by activating glycogen synthase kinase-3β. *J. Alzheimers Dis.* 43 153–165. 10.3233/jad-140198 25079798

[B198] Wang Z. Y. Liu J. G. Li H. Yang H. M. (2016). Pharmacological effects of active components of chinese herbal medicine in the treatment of Alzheimer’s disease: a review. *Am. J. Chinese Med.* 44 1525–1541. 10.1142/S0192415x16500853 27848250

[B199] Weaver C. T. Elson C. O. Fouser L. A. Kolls J. K. (2013). The Th17 pathway and inflammatory diseases of the intestines, lungs, and skin. *Annu. Rev. Pathol. Mech.* 8 477–512. 10.1146/annurev-pathol-011110-130318 23157335 PMC3965671

[B200] Wei S. Peng W. Mai Y. Li K. Wei W. Hu L. (2020). Outer membrane vesicles enhance tau phosphorylation and contribute to cognitive impairment. *J. Cell Physiol.* 235 4843–4855. 10.1002/jcp.29362 31663118

[B201] Weller J. Budson A. (2018). Current understanding of Alzheimer’s disease diagnosis and treatment. *F1000Res* 7:F1000 Faculty Rev-1161. 10.12688/f1000research.14506.1 30135715 PMC6073093

[B202] Whiley L. Chappell K. E. D’hondt E. Lewis M. R. Jiménez B. Snowden S. G. (2021). Metabolic phenotyping reveals a reduction in the bioavailability of serotonin and kynurenine pathway metabolites in both the urine and serum of individuals living with Alzheimer’s disease. *Alzheimers Res. Ther.* 13:20. 10.1186/s13195-020-00741-z 33422142 PMC7797094

[B203] Wilmes L. Collins J. M. O’riordan K. J. O’mahony S. M. Cryan J. F. Clarke G. (2021). Of bowels, brain and behavior: a role for the gut microbiota in psychiatric comorbidities in irritable bowel syndrome. *Neurogastroenterol. Motil.* 33:e14095. 10.1111/nmo.14095 33580895

[B204] Witte A. V. Fobker M. Gellner R. Knecht S. Flöel A. (2009). Caloric restriction improves memory in elderly humans. *Proc. Natl. Acad. Sci. U.S.A.* 106 1255–1260. 10.1073/pnas.0808587106 19171901 PMC2633586

[B205] Wu L. Han Y. Zheng Z. Peng G. Liu P. Yue S. (2021). Altered gut microbial metabolites in amnestic mild cognitive impairment and Alzheimer’s disease: signals in host-microbe interplay. *Nutrients* 13:228. 10.3390/nu13010228 33466861 PMC7829997

[B206] Yang X. Yu D. Xue L. Li H. Du J. (2020). Probiotics modulate the microbiota-gut-brain axis and improve memory deficits in aged SAMP8 mice. *Acta Pharm. Sin. B* 10 475–487. 10.1016/j.apsb.2019.07.001 32140393 PMC7049608

[B207] Yano J. M. Yu K. Donaldson G. P. Shastri G. G. Ann P. Ma L. (2015). Indigenous bacteria from the gut microbiota regulate host serotonin biosynthesis. *Cell* 161 264–276. 10.1016/j.cell.2015.02.047 25860609 PMC4393509

[B208] Yin J. X. Maalouf M. Han P. Zhao M. Gao M. Dharshaun T. (2016). Ketones block amyloid entry and improve cognition in an Alzheimer’s model. *Neurobiol. Aging* 39 25–37. 10.1016/j.neurobiolaging.2015.11.018 26923399

[B209] Yusufov M. Weyandt L. L. Piryatinsky I. (2017). Alzheimer’s disease and diet: a systematic review. *Int. J. Neurosci.* 127 161–175. 10.3109/00207454.2016.1155572 26887612

[B210] Zhai Q. Feng S. Arjan N. Chen W. (2019). A next generation probiotic, *Akkermansia muciniphila*. *Crit. Rev. Food Sci. Nutr.* 59 3227–3236. 10.1080/10408398.2018.1517725 30373382

[B211] Zhan G. Yang N. Li S. Huang N. Fang X. Zhang J. (2018). Abnormal gut microbiota composition contributes to cognitive dysfunction in SAMP8 mice. *Aging* 10 1257–1267. 10.18632/aging.101464 29886457 PMC6046237

[B212] Zhan X. Stamova B. Jin L. W. Decarli C. Phinney B. Sharp F. R. (2016). Gram-negative bacterial molecules associate with Alzheimer disease pathology. *Neurology* 87 2324–2332. 10.1212/wnl.0000000000003391 27784770 PMC5135029

[B213] Zhang C. Yin A. Li H. Wang R. Wu G. Shen J. (2015). Dietary modulation of gut microbiota contributes to alleviation of both genetic and simple obesity in children. *EBioMedicine* 2 968–984. 10.1016/j.ebiom.2015.07.007 26425705 PMC4563136

[B214] Zhang L. Wang Y. Xia X. Y. Shi C. H. Chen W. Song N. (2017). Altered gut microbiota in a mouse model of Alzheimer’s disease. *J. Alzheimers Dis.* 60 1241–1257. 10.3233/Jad-170020 29036812

[B215] Zhang M. Zhao D. Zhou G. Li C. (2020). Dietary pattern, gut microbiota, and Alzheimer’s disease. *J. Agric. Food Chem.* 68 12800–12809. 10.1021/acs.jafc.9b08309 32090565

[B216] Zhang Y. Huang R. R. Cheng M. J. Wang L. R. Chao J. Li J. X. (2019). Gut microbiota from NLRP3-deficient mice ameliorates depressive-like behaviors by regulating astrocyte dysfunction via circHIPK2. *Microbiome* 7:116. 10.1186/S40168-019-0733-3 31439031 PMC6706943

[B217] Zhang Y. X. Li Y. Ma L. N. (2020). Recent advances in research on Alzheimer’s disease in China. *J. Clin. Neurosci.* 81 43–46. 10.1016/j.jocn.2020.09.018 33222956

[B218] Zhao L. Zhang F. Ding X. Wu G. Lam Y. Y. Wang X. (2018). Gut bacteria selectively promoted by dietary fibers alleviate type 2 diabetes. *Science* 359 1151–1156. 10.1126/science.aao5774 29590046

[B219] Zheng Q. Bi R. Xu M. Zhang D. F. Tan L. W. Lu Y. P. (2021). Exploring the genetic association of the ABAT gene with Alzheimer’s disease. *Mol Neurobiol.* 58:1904. 10.1007/s12035-020-02271-z 33483904

[B220] Zhuang Z. Yang R. Wang W. Qi L. Huang T. (2020). Associations between gut microbiota and Alzheimer’s disease, major depressive disorder, and schizophrenia. *J. Neuroinflamm.* 17:288. 10.1186/s12974-020-01961-8 33008395 PMC7532639

[B221] Zhuang Z. Q. Shen L. L. Li W. W. Fu X. Zeng F. Gui L. (2018). Gut microbiota is altered in patients with Alzheimer’s disease. *J. Alzheimers Dis.* 63 1337–1346. 10.3233/Jad-180176 29758946

[B222] Ziegler-Graham K. Brookmeyer R. Johnson E. Arrighi H. M. (2008). Worldwide variation in the doubling time of Alzheimer’s disease incidence rates. *Alzheimers Dement.* 4 316–323. 10.1016/j.jalz.2008.05.2479 18790458

[B223] Zmora N. Bashiardes S. Levy M. Elinav E. (2017). The role of the immune system in metabolic health and disease. *Cell Metab.* 25 506–521. 10.1016/j.cmet.2017.02.006 28273474

[B224] Zolochevska O. Taglialatela G. (2020). Selected microRNAs increase synaptic resilience to the damaging binding of the Alzheimer’s disease amyloid beta oligomers. *Mol. Neurobiol.* 57 2232–2243. 10.1007/s12035-020-01868-8 31997075 PMC7170988

